# Genomic insight and physiological characterization of thermoacidophilic *Alicyclobacillus* isolated from Yellowstone National Park

**DOI:** 10.3389/fmicb.2023.1232587

**Published:** 2023-09-26

**Authors:** Hye Won Kim, Na Kyung Kim, Alex P. R. Phillips, David A. Parker, Ping Liu, Rachel J. Whitaker, Christopher V. Rao, Roderick I. Mackie

**Affiliations:** ^1^Department of Animal Sciences, University of Illinois at Urbana-Champaign, Champaign, IL, United States; ^2^Materials Research Laboratory, Energy and Biosciences Institute, University of Illinois at Urbana-Champaign, Champaign, IL, United States; ^3^Department of Microbiology, University of Illinois at Urbana-Champaign, Champaign, IL, United States; ^4^Westhollow Technology Center, Shell Exploration and Production Inc., Houston, TX, United States; ^5^Carl R. Woese Institute for Genomic Biology, University of Illinois at Urbana-Champaign, Champaign, IL, United States; ^6^Department of Chemical and Biomolecular Engineering, University of Illinois at Urbana-Champaign, Champaign, IL, United States

**Keywords:** *Alicyclobacillus*, thermoacidophilic bacterium, genome sequencing and annotation, Yellowstone Hot Springs, geothermal environment

## Abstract

**Introduction:**

*Alicyclobacillus* has been isolated from extreme environments such as hot springs, volcanoes, as well as pasteurized acidic beverages, because it can tolerate extreme temperatures and acidity. In our previous study, *Alicyclobacillus* was isolated during the enrichment of methane oxidizing bacteria from Yellowstone Hot Spring samples.

**Methods:**

Physiological characterization and genomic exploration of two new *Alicyclobacillus* isolates, AL01A and AL05G, are the main focus of this study to identify their potential relationships with a thermoacidophilic methanotroph (*Methylacidiphilum*) isolated from the same hot spring sediments.

**Results and discussion:**

In the present study, both *Alicyclobacillus* isolates showed optimal growth at pH 3.5 and 55°C, and contain ω-alicyclic fatty acids as a major lipid (*ca.* 60%) in the bacterial membrane. Genomic analysis of these strains revealed specific genes and pathways that the methanotroph genome does not have in the intermediary carbon metabolism pathway such as *serC* (phosphoserine aminotransferase), *comA* (phosphosulfolactate synthase), and DAK (glycerone kinase). Both *Alicyclobacillus* strains were also found to contain transporter systems for extracellular sulfate (ABC transporter), suggesting that they could play an important role in sulfur metabolism in this extreme environment. Genomic analysis of vitamin metabolism revealed *Alicyclobacillus* and *Methylacidiphilum* are able to complement each other’s nutritional deficiencies, resulting in a mutually beneficial relationship, especially in vitamin B_1_(thiamin), B_3_ (niacin), and B_7_ (biotin) metabolism. These findings provide insights into the role of *Alicyclobacillus* isolates in geothermal environments and their unique metabolic adaptations to these environments.

## 1. Introduction

Alicyclobacilli are rod-shaped, gram-positive, spore-forming, and thermo-acidophilic bacteria usually isolated from soil, hot springs, volcanoes, acidic drinks, and equipment from fruit juice manufacturers (Smit et al., 2011; Ciuffreda et al., 2015). In 1992, thermophilic *Bacillus* species were reclassified in a new genus *Alicyclobacillus* based on *16S rRNA* sequence analysis and named for the presence of cyclic-fatty acids as the major natural membrane lipid component (Wisotzkey et al., 1992). These lipids contain fatty acids with a ω-cyclohexane ring that provides stability and integrity to membrane structure at high temperature. They exibit a wide growth temperature range (20°C–70°C) and pH values (0.5–7.5), although their optimum growth is mostly in the acidic region (<pH 4.5) (Ciuffreda et al., 2015). This ability of *Alicyclobacillus* to tolerate extreme environments has made them an attractive target for studying extreme environments and conditions.

Several studies have isolated *Alicyclobacillus* from various extreme environments found in geothermal regions of the world, indicating their adaptability to extreme environments. For example, several species of *Alicyclobacillus acidocaldarius, Alicyclobacillus acidoterrestris, Alicyclobacillus cycloheptanicus, Alicyclobacillus fastidiosus, Alicyclobacillus ferrooxydans, Alicyclobacillus hesperidum, Alicyclobacillus kakegawensis, Alicyclobacillus mali, Alicyclobacillus sendaiensis, Alicyclobacillus shizuokaensis, Alicyclobacillus tegnchongensis, Alicyclobacillus tolerans,* and *Alicyclobacillus vulcanalis* have been isolated from hot soil, solfataric soil, hot spring soil, or geothermal pool samples, with optimum growth temperatures ranging from 40°C to 60°C (Wisotzkey et al., 1992; Albuquerque et al., 2000; Tsuruoka et al., 2003; Simbahan et al., 2004; Karavaiko et al., 2005; Goto et al., 2007; Jiang et al., 2008; Kim et al., 2014; Ciuffreda et al., 2015; Aulitto et al., 2021).

Yellowstone National Park hotspring is a geothermal area with a rich diversity of microorganisms adapted to the extreme conditions of high temperature and acidity. Methanotrophs have been studied in this environment because they play a crucial role in the biological cycling of methane and carbon. During the attempt to isolate methanotrophs from Yellowstone Hot Spring samples (Kim et al., 2022), our research team isolated *Alicyclobacillus* species from Nymph Lake (89.9°C, pH 2.73.) and the Norris Geyser Basin (43.6°C, pH 3.06). After cultivation of the hot spring samples (sediment plus spring water) with 25% CH_4_ and 8% CO_2_ gas composition, tiny colonies were found adjacent to and overlapping with the slow growing methanotroph colonies on the plates that were determined to be *Alicyclobacillus* by *16S rRNA* gene sequencing (Supplementary Figure S1). Since *Alicyclobacillus* is unable to grow on methane as a carbon source, we hypothesized that it could survive in the harsh condition of hotspring sediments by endospore formation and a cross-feeding interaction using a metabolite (s) from methanotrophs.

Physiological and genomic characterization of *Alicyclobacillus* isolates can provide valuable insights into their potential roles in the geothermal environment and their interactions with other microorganisms. Therefore, this study aimed to characterize and explore the genomic features of the two new *Alicyclobacillus* isolates, AL01A and AL05G, and investigate their potential syntrophic relationships with methanotroph isolated from the same hotspring sediment enrichments.

## 2. Materials and methods

### 2.1. Isolation and identification of *Alicyclobacillus*

Samples used in this study were collected from Nymph Lake and Norris Geyser Basin in Yellowstone National Park (Table 1) in September 2017 under permit YELL-2017-SCI-5684. Samples of sediment and spring water were collected from the sites as previously described (Campbell et al., 2017). Briefly, water and sediment designated for culturing were collected in sterile bottles, rinsed once with spring water, and then kept at room temperature while being transported to the laboratory at the University of Illinois at Urbana-Champaign. Samples were delivered to the lab within 1 week of collection. A total of 5 g of sediment and spring water samples were inoculated into the 40 mL of V42 mineral medium at pH 2.0 and incubated at 60°C with 25% CH_4_ and 8% CO_2_ in the headspace to isolate methanotrophs as described in our previous study (Kim et al., 2022). During the isolation and identification steps, mixed colonies with methanotrophs and *Alicyclobacillus*-related species were observed on the V42 agar plates (Supplementary Table S1, V42 mineral medium supplemented with 15 g/L of phytagel). The tiny colonies beside the large methanotroph colonies (Supplementary Figure S1) were isolated and then identified by *16S rRNA* sequencing using the 16S universal primer (27F AGAGTTTGATCCTGGCTCAG, 1492R ACGGCTA CCTTGTTACGACTT). Forward and reverse reads from the amplicons were merged and analyzed with the NCBI nucleotide collection (nr/nt) database.

**Table 1:** Isolation and identification of *Alicyclobacillus* from Yellowstone National Park sediment samples.

Sampling region	Subculture^a^	Morphology on plates	Identification^b^	Designation
Nymph Lake (89.9°C, pH 2.73)	5th transfer	Small, round, and transparent	*A. acidocaldarius* (96.41%)	AL01A
Norris Geyser Basin (43.6°C, pH 3.06)	6th transfer	Small, round, and transparent	*A. acidocaldarius* (97.44%)	AL05G

a Subcultures were performed in the mineral medium V42 (pH 2.5) at 60°C with a subsequent inoculum (25%).

b *Alicyclobacillus* colonies were identified based on the *16S rRNA* sequencing using a universal primer (~1.5 kb sequence length).

### 2.2. Optimum growth condition of the *Alicyclobacillus* isolates

Two *Alicyclobacillus* isolates from Yellowstone National Park (AL01A and AL05G) were maintained individually at −80°C in *Alicyclobacillus* medium (Palop et al., 2000) containing 40% glycerol. *Alicyclobacillus* medium contains 0.2 g/L (NH_4_)_2_SO_4_, 0.5 g/L MgSO_4_·7H_2_O, 0.25 g/L CaCl_2_·2H_2_O, 3 g/L KH_2_PO_4_, 1 g/L yeast extract, and 1 g/L dextrose. In preparation for experiments, each strain was grown in *Alicyclobacillus* medium and adjusted to pH 3.5 for 24 h at 55°C. Effects of temperature (30, 37, 50, 55, 60, and 70°C) and pH (2.0, 2.5, 3.0, 3.5, 4.0, 4.5, and 5.0) on bacterial growth were tested. The pH of the medium was adjusted using HCl or NaOH. Bacterial growth at 0, 2, 4, 6, 8, 10, 12, and 24 h was monitored by measuring the optical density at 600 nm. The growth rate at different pH conditions was calculated using the equation $\mu =\frac{2.303\;\left(\mathrm{lnOD}2-\mathrm{lnOD}1\right)}{\left(T2-T1\right)}$ , where two optical density (OD) values were chosen on the exponential line (OD1 and OD2) and the corresponding time points are *T*1 and *T*2, respectively.

### 2.3. Phenotypic and biochemical characterization

The carbon sources and other chemical utilization and tolerance patterns were determined using the Biolog Microstation system with GEN III microplates. The test panel contains 71 carbon sources, 23 chemical sensitivity assays, and wells for the positive (maximum growth) and negative (no carbon source) controls. Bacterial cultivation and inoculation were performed according to the manufacturer’s instructions with some modifications. For example, bacterial cells were cultivated in the *Alicyclobacillus* medium with the optimized pH adjusted using 0.1 N HCl and temperature conditions (pH 3.5 and 55°C). The inoculum was prepared with inoculum fluid and 100 μL of this inoculum was distributed into each well of the Biolog 96-well microplate and incubated. After incubation, the appearance of positive purple wells was measured using a microplate reader.

### 2.4. Minimum inhibitory concentration determination

Methanol and formate solutions were freshly prepared before each experiment. The minimum inhibitory concentration (MIC) values for AL01A and AL05G were determined by the method currently recommended by Clinical and Laboratory Standards Institute (CLSI). In brief, each microdilution well containing 100 μL of the corresponding two-fold dilution (from 0.039 to 5 mM) was inoculated with 100 μL of a cell suspension (final concentrations of approximately 5.0 × 10^5^ colony forming unit (CFU)/mL). The microdilution trays were incubated at 55°C for 18 h, and the MIC was defined as the lowest concentration of materials for which no visible bacterial growth was observed. In each case, cell suspensions inoculated in the absence of the materials served as a positive control.

### 2.5. Gas chromatography analysis: membrane fatty acid and guaiacol production

Bacterial pellets were collected by centrifugation (12,000 rpm, 1 min) after culture at 55°C for 24 h. Cells were treated with ultrasound using QSonica system (QSonica LLC, CT, United States). Fatty acids were extracted by methanol:chloroform (1:2 v/v) mixture, evaporated under N_2_ to dryness and derivatized into their methyl esters. Fatty acids were converted into their methyl esters and analyzed using a GC-MS system (Agilent Inc., CA, United States) consisting of an Agilent 7890B gas chromatograph, an Agilent 5977A MSD, and ZB-5MS (60 m × 0.32 mm I.D. and 0.25 mm film thickness) capillary column (Phenomenex, CA, United States). The inlet and MS interface temperatures were 250°C, and the ion source temperature was 230°C. An aliquot of 1 mL was injected with the split ratio of 10:1. The helium carrier gas was kept at a constant flow rate of 2.4 mL/min. The temperature program was: 2 min at 150°C, followed by an oven temperature increase of 5°C/min to 300°C. The mass spectrometer was operated in positive electron impact mode (EI) at 69.9 eV ionization energy at *m*/*z* 33–500 scan range.

Guaiacol (2-methoxy phenol) in the supernatants was analyzed using a GC-MS system (Agilent Inc.) consisting of an Agilent 7890B gas chromatograph, an Agilent 5977A MSD, and Innowax-HP (30 m × 0.25 mm I.D. and 0.25 mm film thickness) capillary column (Agilent, United States). The inlet, MS interface, and ion source temperature were 230°C. An aliquot of 2 mL was injected with the split ratio of 10:1. The helium carrier gas was kept at a constant flow rate of 1 mL/min. The temperature program was: 2 min at 70°C, followed by an oven temperature increase of 10°C/min to 250°C and hold for 2 min. The mass spectrometer was operated in positive electron impact mode (EI) at 69.9 eV ionization energy at *m*/*z* 33–500 scan range. Values were evaluated by the Mass Hunter Quantitative Analysis B.08.00 (Agilent Inc.).

### 2.6. DNA extraction, gDNA library preparation, and genome sequencing

Isolated *Alicyclobacillus* colonies were used for the extraction of genomic DNA (gDNA) using a DNeasy^®^ Blood & Tissue Kit (Qiagen, Valencia, CA, United States) according to the manufacturer’s instruction. DNA concentrations were then measured by Qubit with a dsDNA HS assay kit (Life Technologies, Thermo Fisher Scientific Inc., CA, United States). DNA concentrations of AL01A from Nymph Lake and AG05G from Norris Geyser Basin were 3.49 μg/mL (total 698 ng) and 2.81 μg/mL (total 562 ng), respectively. The shotgun gDNA library for the samples was prepared with a Hyper library construction kit (Kapa Biosystem, MA, United States). gDNA libraries were quantitated by qPCR and confirmed by gel electrophoresis. Sequencing was carried out with 251 cycles from each end of the fragments on a NovaSeq 6000 (Illumina, Inc.) using a NovaSeq SP reagent kit (Illumina). Fastq files were generated and demultiplexed with the bcl2fastq v2.20 conversion software (Illumina). A total of 75,261,725 and 59,440,504 paired-end reads with a read size of 250 × 2 nt were obtained for AL01A and AL05G, respectively (Table 2). The Phred quality-scores used an ASCII offset of 33 known as Sanger scores.

**Table 2:** Whole genome sequencing of *Alicyclobacillus* isolates from Yellowstone National Park.

Genomic statistics	AL01A	AL05G
Total raw sequences	75,261,725	59,440,504
Phread score	>33	>33
After *de novo* assembly^a^		
Contigs	89	339
Bases	3,187,819 bp	3,660,934 bp
GC content (%)	62.1%	61.8%
CDS	3,121	3,598
Alignment rate against *A. acidocaldarius*^b^	78.63%	76.61%
Alignment rate against *A. sendaiensis*^c^	74.74%	71.31%

a *De novo* assembly was performed with Spades.

b *A. acidocaldarius* DSM 446 genome (GenBank assembly accession number: GCA_000024285.1).

c *A. sendaiensis* NBRC 100866 genome (GenBank assembly accession number: GCA_001552675.1).

### 2.7. Genome assembly

Since the initial coverage depth of each sample was too high (9,845 and 12,466 for AL01A and AL05G, respectively), Seqtk was used to adjust the coverage depth to 100×. Processed paired-end genome sequencing reads were subject to *de novo* metagenome assembly using MetaSpades (ver. 3.14.1). Contigs shorter than 1 kb were dropped from the pool. Original reads were mapped to the contigs using BWA (ver. 0.7.17) and the read coverage of each contig was calculated. Contigs were identified based on the presence of repeated sequences on both ends using the previously described protocol (Jørgensen et al., 2014).

### 2.8. Comparative genome analysis

The genomes were mapped to *A. acidocaldarius* and *A. sendaiensis* reference genomes using BWA. The average nucleotide identity (ANI) was also calculated by comparing the sequences with other strains. All available genome sequences in RefSeq database (National Center for Biotechnology Information, NCBI) were adopted as reference genomes and genomic data were obtained from the NCBI site. Pyani (ver. 0.2.10) was used to generate the phylogeny from the comparative analysis of the ANI values.

### 2.9. Metabolic pathways and relative gene alignment analysis

Open reading frames on the assembled contigs (89 and 339 contigs each) were identified and translated into amino acid sequences using PROKKA (ver. 1.14.6). Taxonomic and functional annotations were performed by searching all potential amino acid sequences against KEGG and EggNOG. Clusters of orthologous group (COG) functional categories of the annotated genes were characterized by eggNOG-mapper (ver. 2.0) analysis. Antimicrobial resistance genes were identified based on the comprehensive antibiotic resistance database (CARD; https://card.mcmaster.ca).

### 2.10. Nucleotide sequence accession numbers and data availability

The assembled genomes have been deposited at the NCBI under submission number SUB13701364 (BioProject number: PRJNA997735; BioSample number: SAMN36688594; Accession number: JAUPJT000000000) and SUB13702191 (BioProject number: PRJNA997737; BioSample number: SAMN36688642; Accession number: JAUPJS000000000) for AL01A and AL05G, respectively. The version described in the manuscript is the first version.

### 2.11. HPLC analysis: metabolite production

Bacterial supernatants were collected by centrifugation (12,000 rpm, 1 min) after culture at 55°C for 24 h and then filtered with syringe filters (0.2 μm). Lactate, acetate, succinate, glucose, citrate, pyruvate, formate, and ethanol were measured using a Shimadzu LC-20AD Series HPLC system (Shimadzu Corporation, Kyoto, Japan) consisting of Shimadzu LC-20AD HPLC pump, Shimadzu series DGU-20A5R Degasser, and a Shimadzu SIL-10AF autosampler. Samples were injected into an Aminex HPX-87P column (Bio-Rad Laboratories, Hercules, CA, United States) and then detected with Shimadzu RID-10A refractive index detector and SPD-20A UV/VIS detector (Shimadzu Corporation).

## 3. Results

### 3.1. Optimum bacterial growth conditions of *Alicyclobacillus* strains isolated from Yellowstone Hot Spring samples

**Figure 1:**
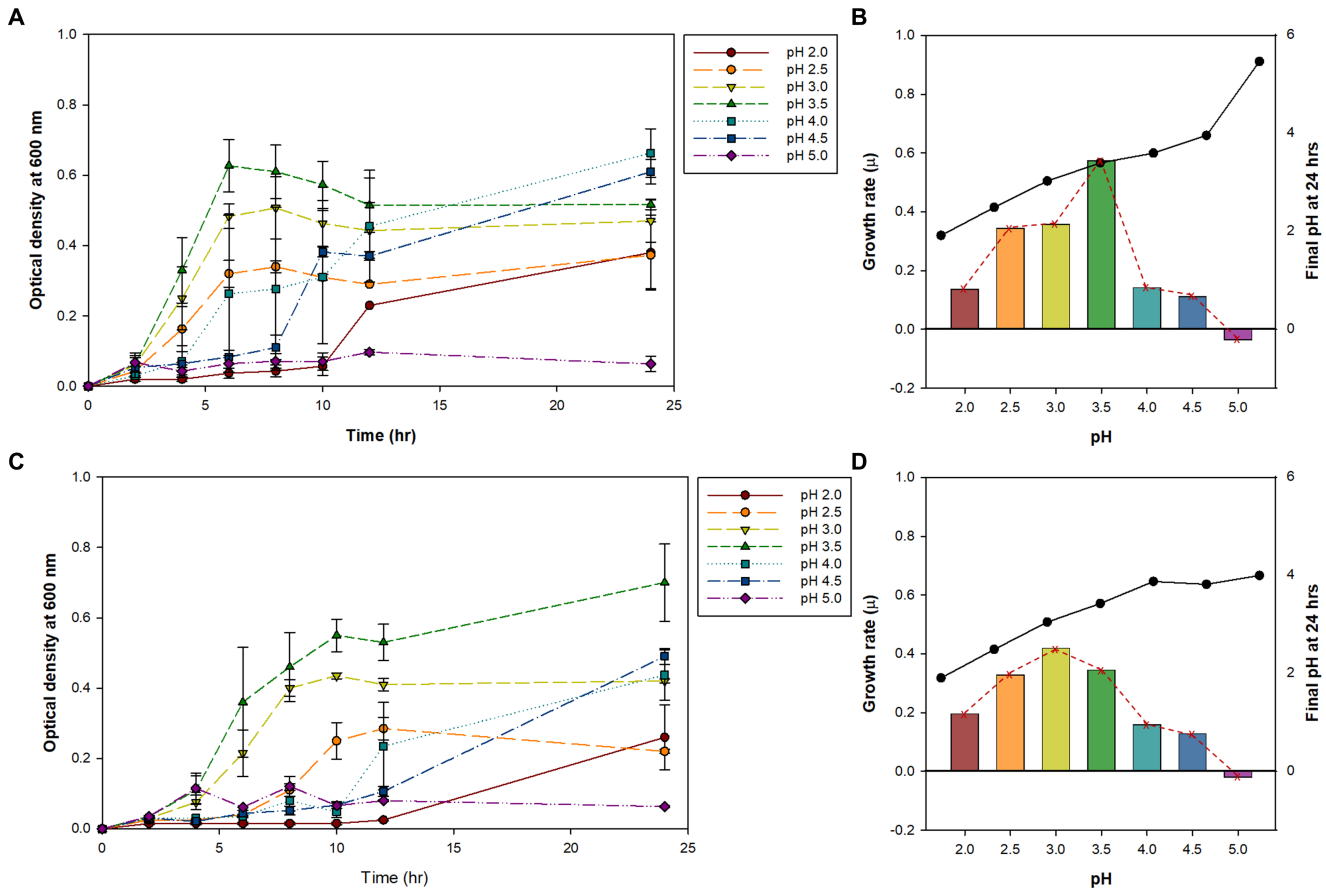
**(A,C)** The bacterial growth curve of *Alicyclobacillus* strains AL01A and AL05G at 55°C with different pH ranging from 2.0 to 5.0. **(B,D)** Maximum growth rate (*μ*) and final pH at 24 h represented as bars with red dotted line and black solid line graphs, respectively. **(A,B)** AL01A, **(C,D)** AL05G.

Previously, we isolated small, round, and transparent colonies during the isolation of methanotrophs from Yellowstone Hot Spring samples (Kim et al., 2022). The *16S rRNA*-based PCR of the isolates identified *Alicyclobacillus* species from Nymph Lake and Norris Geyser Basin samples with 96.41% and 97.44% identity to *A. acidocaldarius*, respectively (Table 1). We named them AL01A and AL05G. Strains AL01A and AL05G had optimum growth temperatures (about 55°C) that were very similar to the other reference strains. Both strains could grow at 60°C but showed a reduced growth rate compared to 55°C and could not grow at 37°C. The growth curves of bacteria cultured in *Alicyclobacillus* medium at 55°C in a pH range from pH 2.0 to 5.0 are presented in Figure 1. In general, *Alicycobacillus* isolates grew rapidly and reached high optical density at pH 3.5 followed by pH 3.0 and pH 2.5. Bacterial growth was inhibited when pH was less than 2.0 or more than 4.5 with the optical density under these pH conditions at 6 h ranged from 0.005 to 0.08 while the optical density at pH 3.5 reached 0.63 and 0.36 in AL01A and AL05G, respectively. The growth rate (*μ*) of each isolate is shown in Figures 1B,D. The maximum growth rate of strain AL01A was 0.57 h^−1^ at pH 3.5, while that of strain AL05G was 0.42 h^−1^ at pH 3.0. As both strains grew well and showed maximum optical density at pH 3.5, this was determined to be the optimum pH condition for growth. The final pH of the cultures at 24 h generally decreased compared to the initial pH conditions except for the AL01A cultures at pH 5.0 (final pH: 5.45).

### 3.2. Carbon source utilization and chemical sensitivity

**Figure 2:**
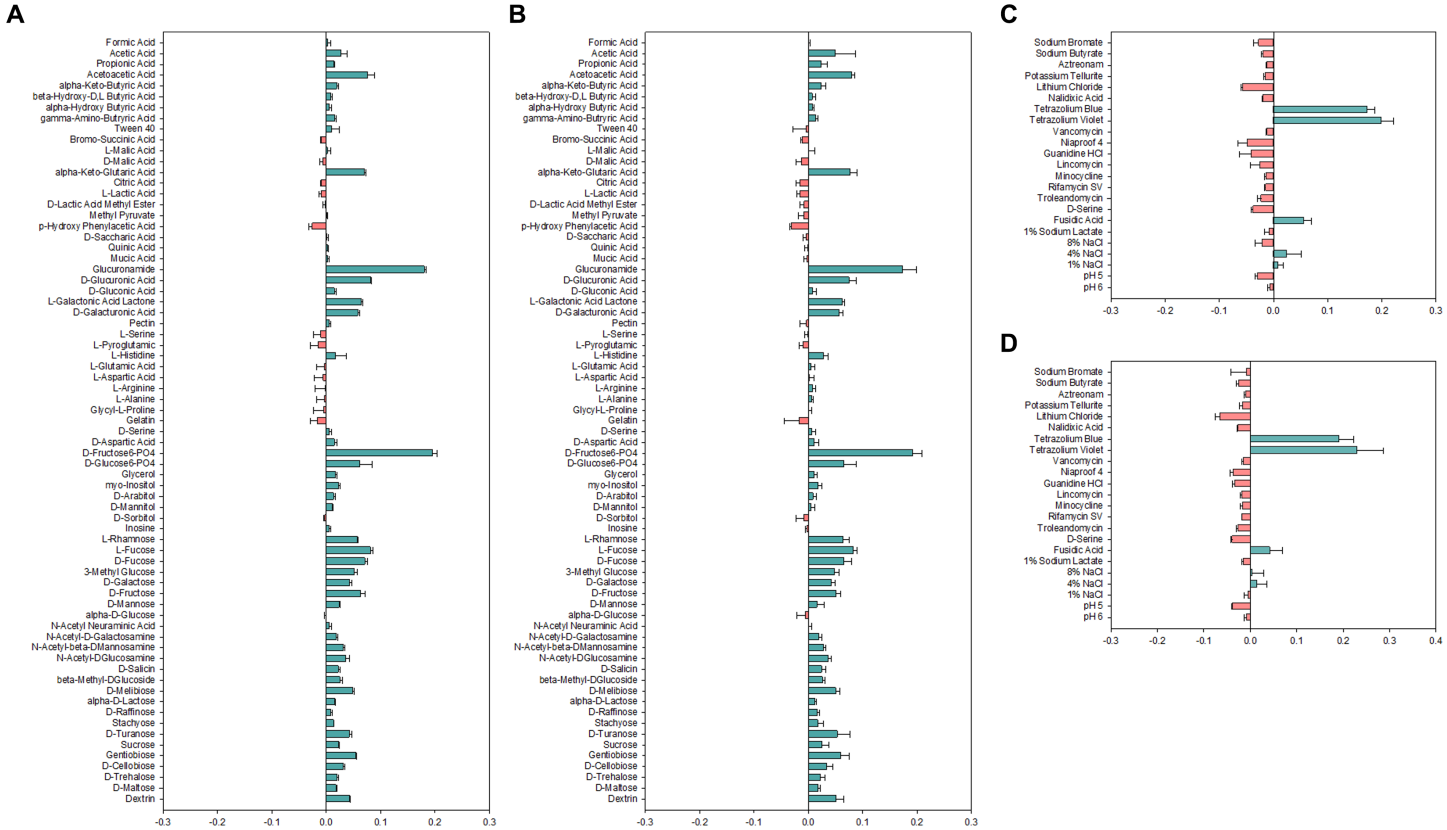
**(A,B)** Carbon source utilization and **(C,D)** chemical sensitivity test of **(A,C)** AL01A and **(B,D)** AL05G. The green and red bars indicate positive and negative reactions, respectively. The measurements were taken using optical density at 600 nm and subsequently normalized to the control value.

Carbon sources used for growth are important determinants for identification and classification of bacteria. Therefore, carbon source utilization of *Alicyclobacillus* strains was assessed by employing the Biolog GEN III MicroPlate system to obtain an overview of metabolic profiles (Figure 2). Both strains AL01A and AL05G were shown to have high capability to use D-fructose-6-PO_4_, D-glucose-6-PO_4_, L-rhamnose, fucose, 3-methyl glucose, D-galactose, D-fructose, and D-mannose. Both strains were unable to use p-hydroxy phenylacetic acid as a carbon source. There was no growth observed on amino acids such as L-serine, L-pyroglutamic acid, L-glutamic acid, L-aspartic acid, L-arginine, L-alanine, and glycyl-L-proline. The system also showed that the growth of *Alicyclobacillus* strain AL01A and AL05G was not inhibited by tetrazolium blue, tetrazolium violet, fusidic acid, and 4% NaCl.

### 3.3. Identification of *Alicyclobacillus* strains using genomic information

**Figure 3:**
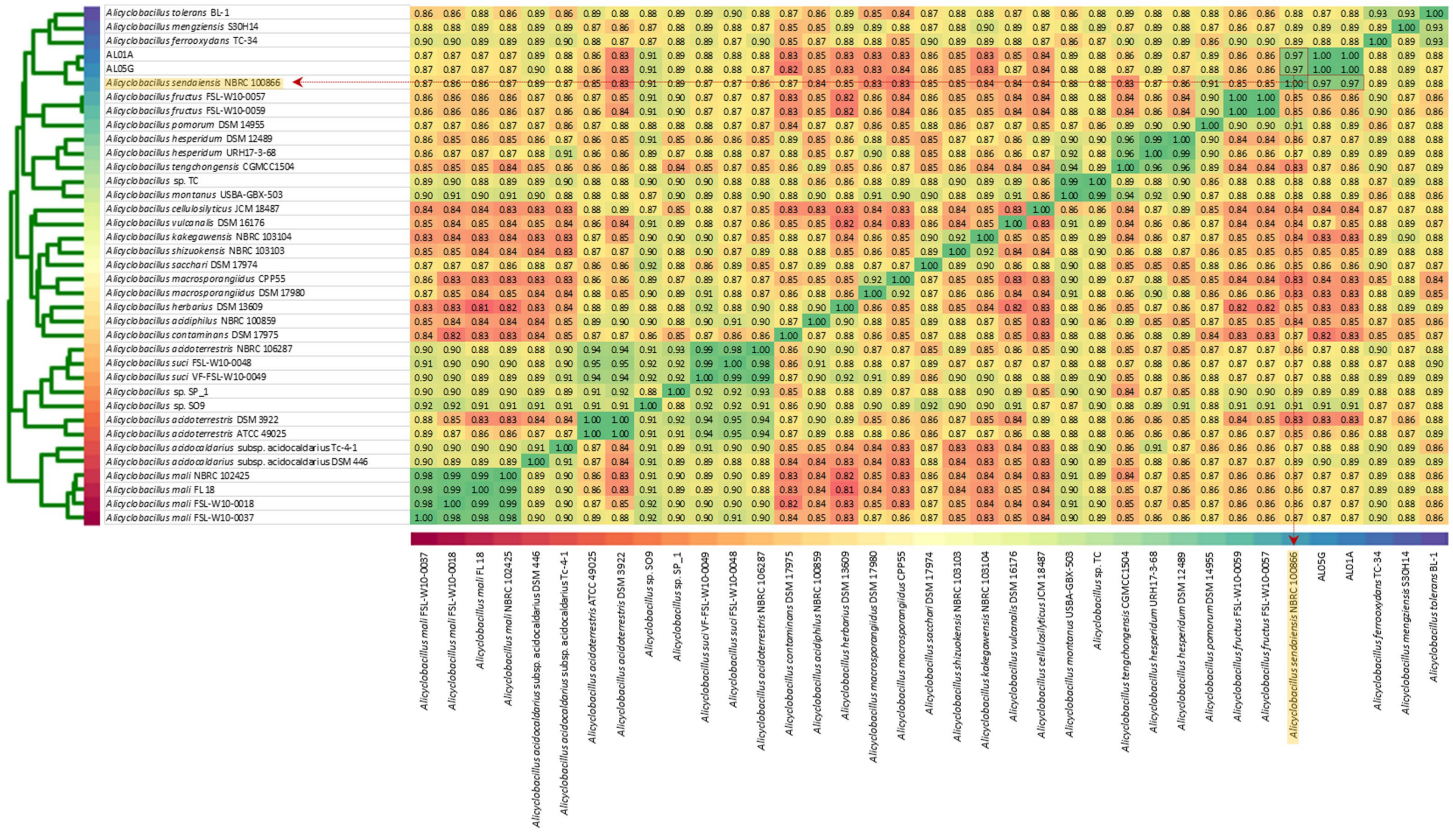
Average nucleotide identity among AL01A, AL05G, and 35 reference *Alicyclobacillus* strains. Reference strains include *A. acidocaldarius* DSM 446 and *A. sendaiensis* NBRC 100866) but also other *Alicyclobacillus* species registered in the NCBI database (*A. tolerance* BL-1, *A. mengziensis* S30H14, *A. ferroxydans* TC-34, *A. fructus* FSL-W10-0057, *A. fructus* FSL-W10-0059, *A. pomorum* DSM 14955, *A. hesperidum* DSM 12489, *A. hesperidum* URH17-3-68, *A. tengchongensis* CGMC C1504, *Alicyclobacillus* sp. TC, *A. montanus* USBA-GBX-503, *A. cellulosilyticus* JCM 18487, *A. vulcanalis* DSM 16176, *A. kakegawensis* NBRC 103104, *A. shizuokensis* NBRC 103103, *A. sacchari* DSM 17974, *A. macrosporangiidus* CP P55, *A. macrosporangiidus* DSM 17980, *A. herbarius* DSM 13609, *A. acidiphilus* NBRC 100859, *A. contaminans* DSM 17975, *A. acidoterrestris* NBRC 106287, *A. suci* FSL-W10-0048, *A. suci* VF-FSL-W10-0049, *Alicyclobacillus* sp. SP_1, *Alicyclobacillus* sp. SO9, *A. acidoterrestris* DSM 392, *A. acidoterrestris* ATCC 49025, *A. acidocaldarius* subsp. Acidocaldarius TC-4-1, *A. mali* NBRC 102425, *A. mali* FL18, *A. mali* FSL-W10-0018, and *A. mali* FSL-W10-0037. A heatmap was produced utilizing relative values ranging from 0.81 (red) to 1.00 (green).

The two isolates had the following genomic features confirmed by the whole genome sequencing: number of contigs (89 and 339 contigs, respectively), total sequence length (3.2 and 3.7 Mbp), GC content (62.1% and 61.8%), and coding DNA sequence (3,121 and 3,598 CDS) (Table 2). For comparison, ANI values were also calculated not only with two species shown in *16S rRNA*-based PCR with % identity (*A. acidocaldarius* DSM 446 and *A. sendaiensis* NBRC 100866) but also 35 other *Alicyclobacillus* species deposited in the NCBI database. In contrast to the *16S rRNA* PCR results, the AL01A and AL05G genomes showed the highest ANI values (0.97) against *A. sendaiensis* NBRC 100866, while their ANI values against *A. acidocaldarius* DSM 446 was 0.86 (Figure 3). When the sequencing reads of each isolate were mapped to the reference *A. sendaiensis* genome, the overall alignment rate was 74.74% and 71.31%, respectively for the two strains.

### 3.4. Minimum inhibitory concentration

**Figure 4:**
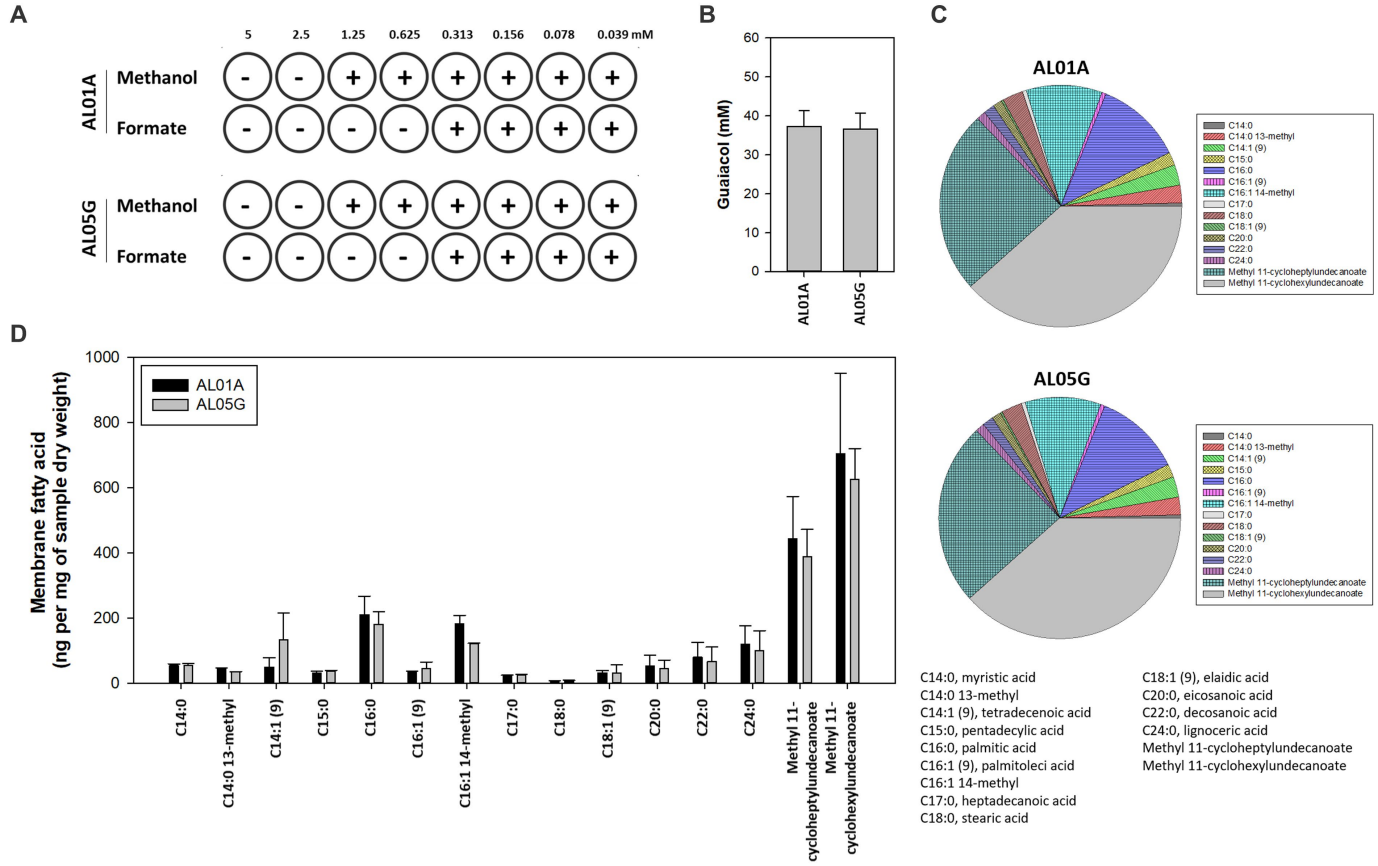
**(A)** The minimum inhibitory concentration of methanol and formate for *Alicyclobacillus* isolates AL01A and AL05G in the presence of methanol and formate. **(B)** Guaiacol production of AL01A and AL05G. **(C,D)** Membrane fatty acid composition of AL01A and AL05G. The pie graph indicates the relative composition of the membrane fatty acids, while the bar graph indicates quantitative fatty acids. All experiments were performed in triplicate.

The susceptibility of AL01A and AL05G cultivated at pH 3.5 at 55°C was assessed against methanol and formate (0.04 to 5 mM) using the broth microdilution method (Figure 4A). Minimum inhibitory concentrations (MICs) against methanol and formate were 1.25 mM and 0.31 mM, respectively, in both strains.

### 3.5. Membrane fatty acid composition

The membrane fatty acid composition of two isolates is shown in Figures 4C,D. A total of 15 different fatty acids were found in the bacterial membrane in this study. The 11 predominant fatty acids identified were C14:0 13-methyl, tetradecenoic acid [C14:1 (cis-9)], pentadecylic acid (C15:0), palmitic acid (C16:0), C16:1 14-methyl, stearic acid (C18:0), eicosanoic acid (C20:0), decosanoic acid (C22:0), lignoceric acid (C24:0), methyl 11-cycloheptylundecanoate, and methyl 11-cyclohexylundecanoate; while the other four minor fatty acids were myristic acid (C14:0), palmitoleic acid [C16:1 (trans-9)], heptadecanoic acid (C17:0), eladic acid [C18:1 (trans-9)] for which composition values were below 1%.

The composition of ω-alicyclic fatty acids in AL01A and AL05G membranes was approximately 60% (62.89% and 59.90%, respectively). The isolates showed the highest amount of methyl 11-cyclohexylundecanoate (704.46 and 625.81 ng per mg of AL01A and AL05G samples dry weight, respectively) followed by methyl 11-cycloheptylundecanoate (443.92 and 388.92 ng per mg of samples dry weight).

### 3.6. Clusters of orthologous groups

**Figure 5:**
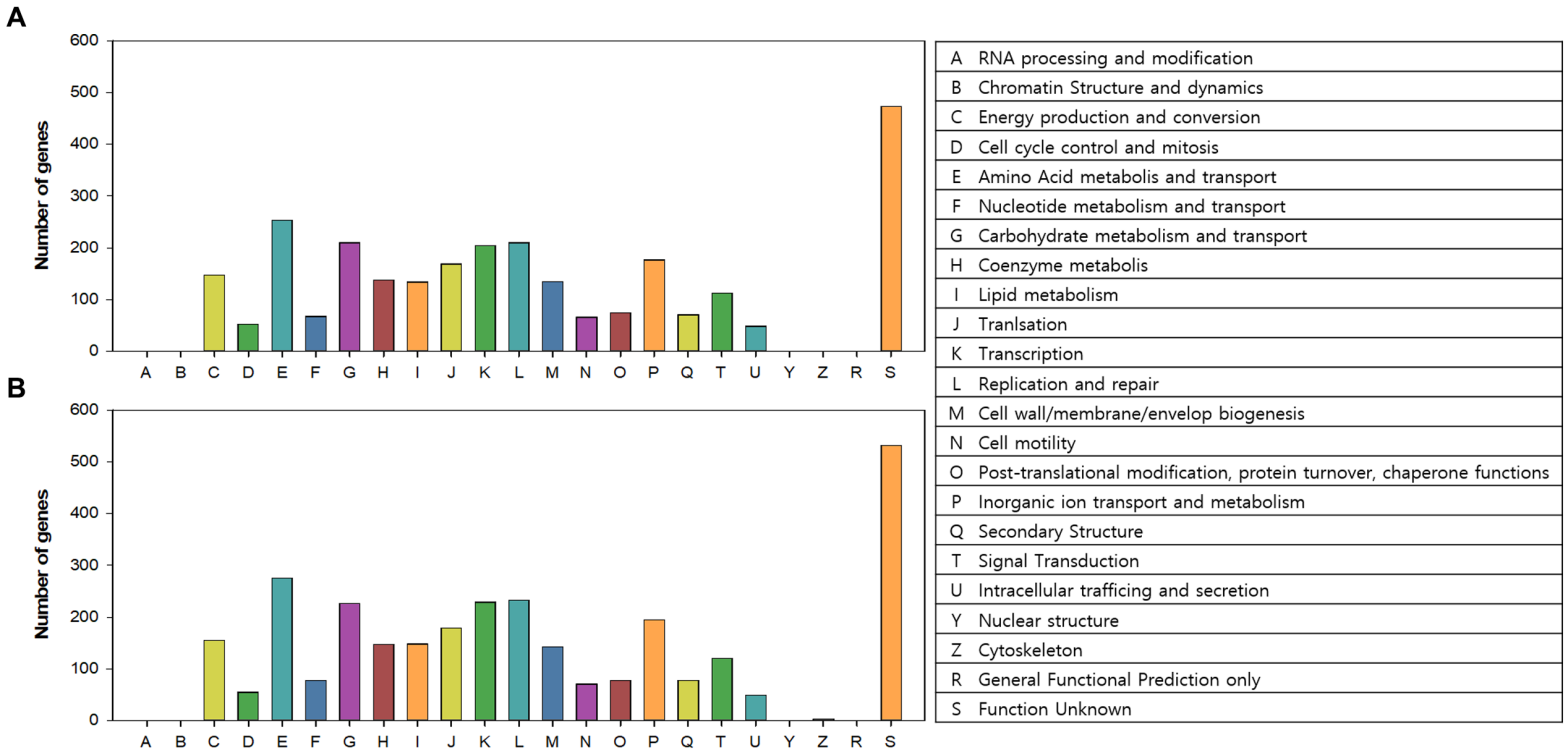
Clusters of orthologous groups (COGs) category of the **(A)** AL01A and **(B)** AL05G genomes analyzed by EggNog mapper.

COG categories of the annotated genes (2,615 and 2,866 genes in AL01A and AL05G, respectively) are shown in Figure 5. Approximately 17% of the annotated genes in both genomes were of unknown function. The highest portion of annotated genes belonged to amino acid metabolism and transport (9.3% and 9.2%, respectively) followed by carbohydrate metabolism and transport (7.7% and 7.6%), replication and repair (7.7% and 7.8%), and transcription (7.4% and 7.7%).

### 3.7. Genes associated with energy, nitrogen and sulfur metabolism

For comparison, genes associated with energy metabolism in the two *Alicyclobacillus* isolates, *A. acidocaldarius* DSM 446, and *Methylacidiphilum* YNP IV genome were analyzed. In Supplementary Figure S3–S5, the presence of the gene in each bacterial genome assembly was indicated by color in the KEGG metabolic pathway (red: AL01A, orange: AL05G, yellow: *A. acidocaldarius* DSM 446, green: YNP IV). Most genes related to intermediary carbon metabolism in *Alicyclobacillus* isolates AL01A and AL05G overlapped with that in *Methylacidiphilum* YNP IV (Supplementary Figure S3). The exceptions are the genes encoding phosphoserine aminotransferase (*serC*) and phosphosulfolactate synthase (*comA*) that are not found in the *Methylacidiphilum* YNP IV genome. The gene encoding glycerone kinase (DAK; also known as Dihydroxyacetone kinase) was only found in AL01A genome, while it was not present in the AL05G and *A. acidocaldarius* DSM 446 genome.

Genes associated with nitrogen metabolism showed that *Alicyclobacillus* isolates have no system for denitrification, nitrogen fixation, and nitrification while the methanotroph *Methylacidiphilum* YNP IV was shown to have these systems based on the genome information (Supplementary Figure S4). Instead, *Alicyclobacillus* isolates seem to play an important role in sulfur metabolism. Both AL01A and AL05G have transporter systems for the extracellular sulfate (ABC transporter) (Supplementary Figure S5). Compared to the *A. acidocaldarius* DMS 446 genome, however, our isolates do not have genes associated with the alkane sulfonate transporter system found in *Methylacidiphilum* YNP IV.

### 3.8. Antimicrobial resistance-related genes

The Resistance Gene Identifier (RGI) algorithmically predicts antimicrobial resistance genes from submitted genomes.^1^ A strict RGI match is not identical to the reference protein sequence but the bit-score of the matched sequence is greater than the BLASTP bit-score cutoff, while loose RGI matches have a bit-score less than the BLASTP bit-score cut-off. Among the antimicrobial resistance genes in both AL01A and AL05G, three strict hits on small multidrug resistance (SMR) antibiotic efflux pump, vanT, and glycopeptide resistance gene cluster, were observed (Supplementary Tables S2 and S3). The top three antimicrobial resistance gene families by number among loose hits were the ATP-binding cassette (ABC) antibiotic efflux pump, major facilitator superfamily (MFS) antibiotic, and resistance-nodulation-cell division (RND) antibiotic efflux pump.

### 3.9. Vitamin metabolism

**Figure 6:**
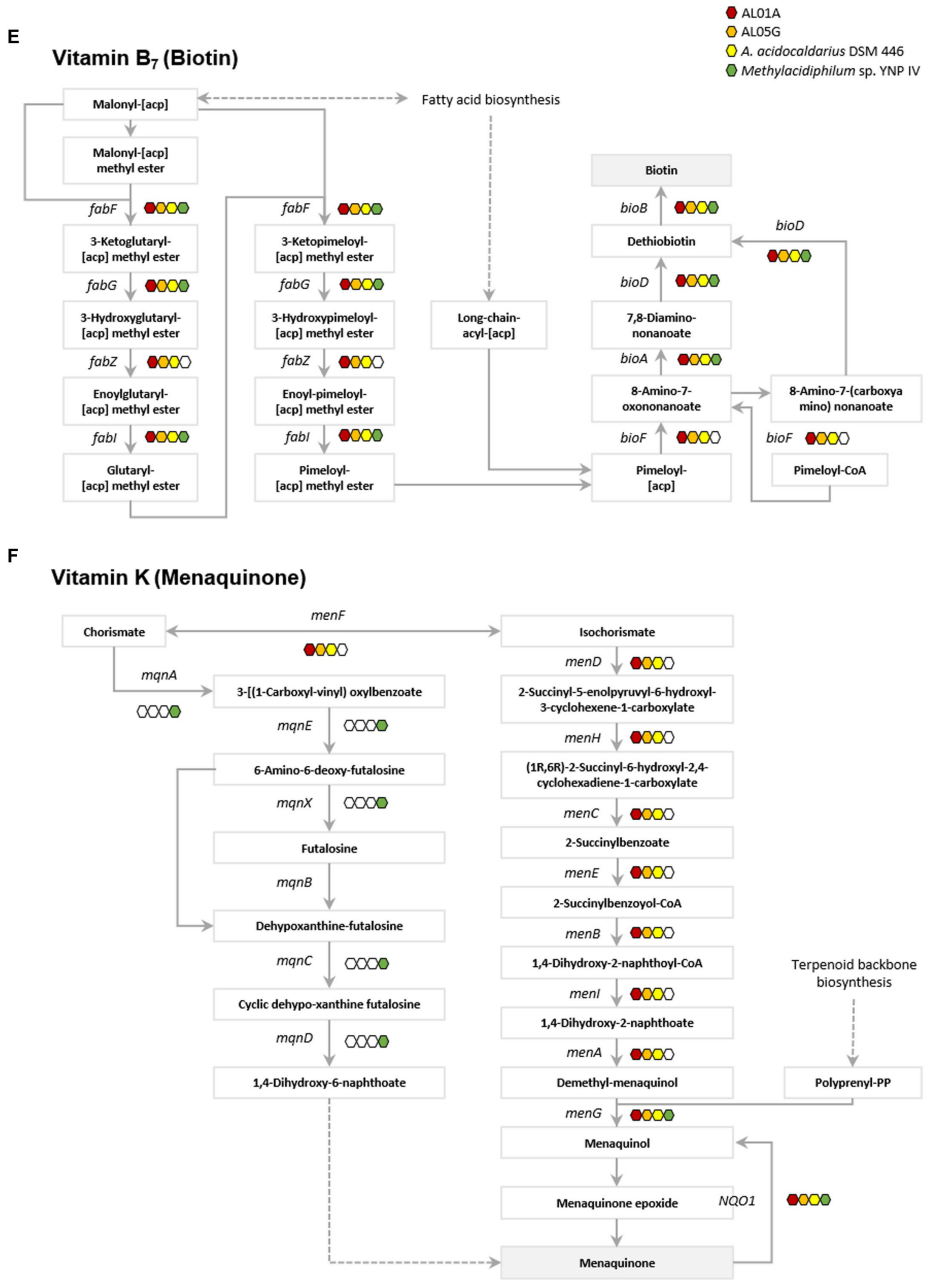
Presence of genes associated with vitamin metabolism. **(A)** Vitamin B1 (thiamin), **(B)** vitamin B3 (nicotinic acid), **(C)** vitamin B5 (pantothenate), **(D)** vitamin B6 (pyridoxine), **(E)** vitamin B7 (biotin), and **(F)** vitamin K (metaquinone). Red, orange, yellow, and green indicate the presence of genes in the AL01A, AL05G, *A. acidocaldarius* DSM 446, and YNP IV genome. White indicates the absence of genes.

Genes involved in vitamin metabolism in the two *Alicyclobacillus* isolates, *A. acidocaldarius* DSM 446 and *Methylacidiphilum* YNP IV are depicted in Figure 6; Supplementary Figures S5–S16. Through genome analysis, it was revealed that *Alicyclobacillus* utilizes cysteine and glycine in vitamin B_1_ (thiamin) metabolism, whereas *Methylacidiphilum* YNP IV employs the tyrosine biosynthetic pathway for this purpose (Supplementary Figure S5). *Alicyclobacillus* also possesses the *enaC* gene, which encodes for thiamin monophosphate phosphohydrolase, an enzyme responsible for the production of thiamine as the final product (Figure 6A). Furthermore, it was found that both *Alicyclobacillus* and *Methylacidiphilum* YNP IV utilize different genes in the process of NAD^+^ and NADP^+^ synthesis (*nad E* and *ppnK* in *Alicyclobacillus*; *NADSYN1* and *NNT* in *Methylacidiphilum* YNP IV) for vitamin B_3_ (nicotinic acid) metabolism. Although all the tested genomes contain the genes for producing nicotinate (*pncB*), only *Alicyclobacillus* genomes have the genes responsible for mediating intermediate production (*punA, ushA,* and *SIR2*) (Figure 6B).

The *Methylacidiphilum* YNP IV genome was found to possess genes (*preT* and *dht*) responsible for utilizing uracil to produce β-alanine in vitamin B_5_ metabolism, while *Alicyclobacillus* could potentially produce β-alanine from L-aspartate or 3-aminopropanal *via panD* or *ALDH* gene expression, respectively (Figure 6C). Regarding vitamin B_6_ metabolism, only the *Methylacidiphilum* YNP IV genome was found to contain *pdxH*, a gene that plays a critical role in pyridoxamine oxidoreductase activity. Though *Alicyclobacillus* lacks the *pdxH* gene, it could utilize glutamine and metabolites from pentose phosphate pathway (D-ribose-5-phospate) and glycolysis (D-glyceraldehyde 3-phospate) to produce vitamin B_6_ through the expression of the *pdxS* gene (Figure 6D). In vitamin B7 (biotin) metabolism, it was found that both *Alicyclobacillus* and *Methylacidiphilum* YNP IV possess most of the *fab* and *bio* genes involved in biotin production. However, only the *Alicyclobacillus* genome contains *fabZ* and *bioF*, which connects the pathway for biotin production. Regarding vitamin K (menaquinone) metabolism, both bacterial strains utilize different pathways to produce menaquinone. *Methylacidiphilum* YNP IV employs th*e mqn* cluster, while *Alicyclobacillus* uses the *men* cluster.

### 3.10. Metabolites

**Figure 7:**
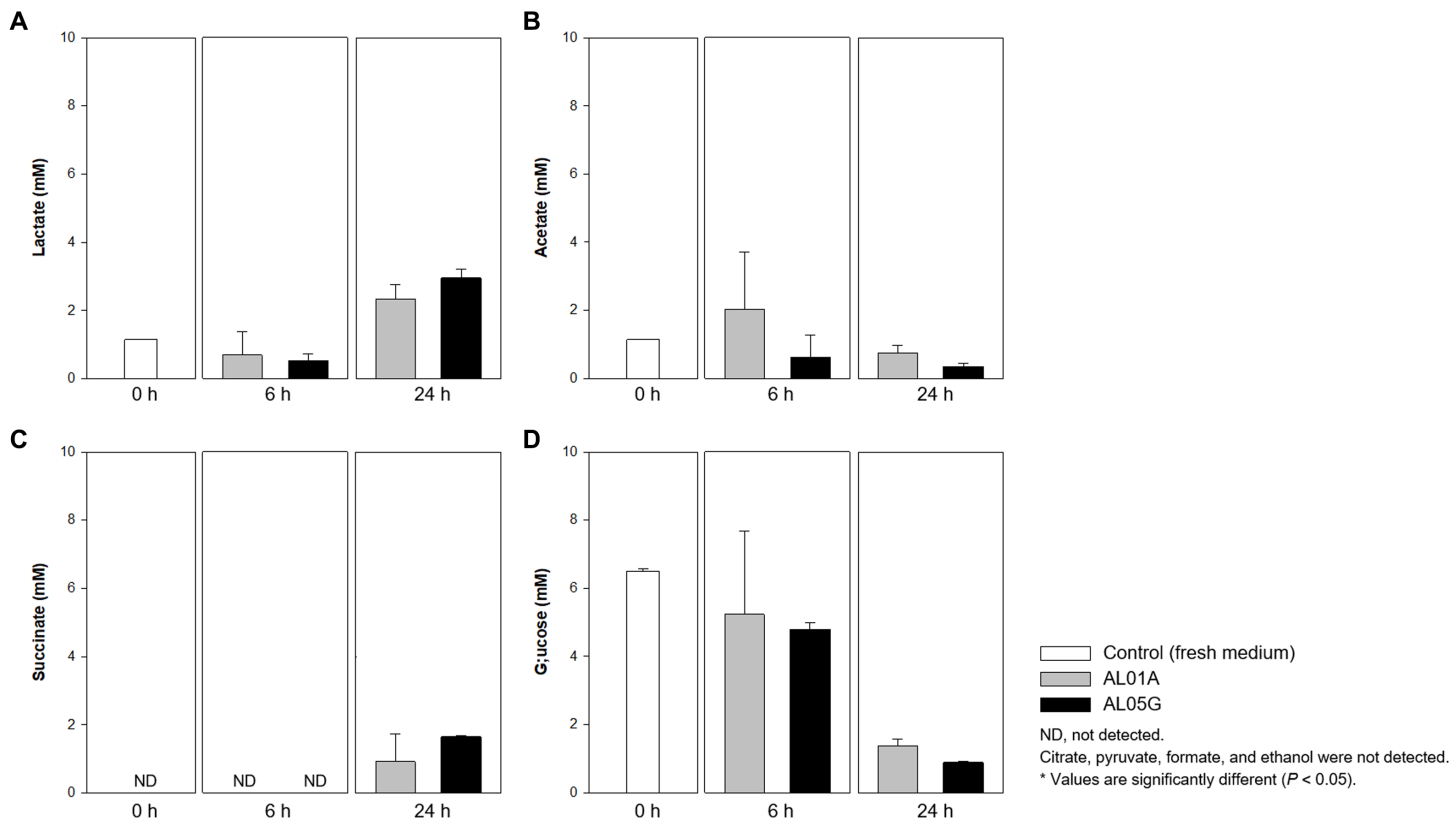
Levels of **(A)** lactate, **(B)** acetate, **(C)** succinate, and **(D)** glucose produced or utilized by *Alicyclobacillus* strain AL01A and AL05G incubated at pH 3.5 and 55°C for 6 and 24 h. Citrate, pyruvate, formate, and ethanol were not detected by the HPLC method used.

Guaiacol (2-methoxy phenol) production is a common characteristic of the genus *Alicyclobacillus*, although the amount of this compound is variable and some strains are unable to produce guaiacol. In the current study, the isolated *Alicyclobacillus* species from Yellowstone National Park could produce approximately 37 pM of guaiacol (Figure 4B). HPLC analysis of the supernatants from 6 and 24 h cultures is shown in Figure 7.

Both *Alicyclobacillus* isolates utilize glucose in the medium (initial 6.49 mM) and produce lactate and succinate. The final level of lactate and succinate reached 2.34–3.00 and 0.93–1.66 mM, respectively. Citrate, pyruvate, formate, and ethanol were not detected by the HPLC method used.

## 4. Discussion

Isolating pure cultures of certain microorganisms from complex microbial consortia requires special cultivation strategies based on an understanding of growth requirements, metabolic characteristics and microbial interactions. Commonly, unintentional isolation of untargeted microorganisms or contamination of DNA occurs during cultivation for the purpose of microbial isolation (Kawanishi et al., 2011), especially from extreme environments with low microbial diversity (Delavat et al., 2012; Böhnke and Perner, 2022; Kim et al., 2022). In our study, *Alicyclobacillus* was inadvertantly co-cultured during the original isolation of a methanotroph from the Yellowstone Hot Spring samples. Tiny *Alicyclobacillus* and relatively larger *Methylacidophylum* YNP IV colonies grew closely together during growth on plates, which makes them difficult to separate in pure culture. During the resuscitation of the methanotroph isolates from glycerol stocks under the relaxed growth conditions at pH 3.5 and 55°C, *Alicyclobacillus* overgrew the methanotroph culture, and then was easily isolated as a pure culture. We describe herein the isolation of the thermoacidophilic bacteria, *Alicyclobacillus* AL01A and AL05G, from Yellowstone Hot Spring samples and its physiological and genomic characterization.

The first *Alicyclobacillus* species was isolated from hot springs in Tohoku district in Japan in 1967 and was named *Bacillus acidocaldarius* (Uchino and Doi, 1967). Similar bacteria were isolated from sediments in thermoacidophilic samples obtained from hotsprings in Yellowstone National Park (Darland and Brock, 1971). Interestingly, these are the same sites that we have been sampling only 50 years later. Several findings of thermo-acidophilic *Bacillus* strains, however, showed that they were distinct from other *Bacillus* species, which led to the proposal of a new genus, *Alicyclobacillus* (Oshima and Ariga, 1975; Hippchen et al., 1981; Wisotzkey et al., 1992; Tourova et al., 1995; Durand, 1996). Most *Alicyclobacillus* species optimally grow from 40°C to 55°C temperature range, while some thermotolerant species could grow at temperatures up to 70°C. Most species can grow from pH 2.0 to 6.0 and they also contained ω-cyclohexane fatty acids as the major components in their membranes (up to 65%) (Ciuffreda et al., 2015). In the present study, the new *Alicyclobacillus* spp. from the Yellowstone Hot Springs showed optimum growth at pH 3.5 and 55°C. This is in agreement with other studies on *Alicyclobacillus* strains that also demonstrated optimum growth conditions ranging from pH 3.0 to 3.5 and 55°C to 60°C (Chang and Kang, 2004; Ciuffreda et al., 2015). The composition of ω-alicyclic fatty acids was approximately 60% similar to the other *Alicyclobacillus* species. Guaiacol (2-methoxy phenol) production is a common characteristic of this genus, although not all Alicyclobacilli produce guaiacol that causes a characteristic disinfectant-like odor or flavor. It was reported that there was a significant variation in guaiacol production among *A. acidoterrestris* strains isolated from commercial fruit crop soils (Ciuffreda et al., 2015). Based on the GC-MS analysis, our isolates were confirmed to produce guaiacol.

The first completed genome sequence of the family Alicyclobacillaceae was reported in 2010 (Mavromatis et al., 2010). *A. acidocaldarius* strain DSM 446 had a 3.2 Mbp genome with 61.9% of G + C content and with 3,153 protein-coding genes. Approximately 32% of the annotated genes were of unknown function. The number of genes associated with the general COG functional categories were highest in general function prediction only (8.4%) followed by carbohydrate transport and metabolism (6.4%), and amino acid transport and metabolism (6.4%). The *16S rRNA* amplicon sequencing showed that the *Alicyclobacillus* isolates of this study showed 96.4% to 97.4% identity against *A. acidocaldarius*. Average nucleotide identity values were, however, highest against *A. sendaiensis* NBRC 100866. The genomic features confirmed by the whole genome sequencing were 3.2–3.7 Mbp, 61.8%–62.1% of G + C contents, and 3,121–3,598 protein-coding genes similar to the reference genome. Otherwise, the COG functional categories of the genome were different from the reference that the highest number was shown in the amino acid metabolism and transport (*ca.* 9%) except for the genes of unknown function.

Despite the AL01A and AL05G genome including many genes related to amino acid metabolism and transport, the C source utilization test showed a negative reaction, i.e., no growth on amino acids including L-serine, L-pyroglutamic, L-glutamic acid, L-aspartic acid, L-arginine, L-alanine, and glycyl-L-proline. It is possible that these genes are not expressed under these specific growth conditions. Nevertheless, the growth of *Alicyclobacillus* is reliant on NH_4_ as a source of nitrogen. The results of nutrient utilization tests demonstrated that both isolates also utilized histidine and serine as N and C sources for growth. Otherwise, most of the C source tested can be used by the two *Alicyclobacillus* isolates. HPLC data showed that the isolates could produce lactate, acetate, and succinate by metabolizing glucose (Figure 6) consistent with the presence of these genes and pathways in the genome of the isolates. To test the activity of each gene during the metabolism, however, transcriptional studies will be required according to the nutrient conditions in a further study.

The unique characteristic of *Alicyclobacillus* species is the presence of ω-alicyclic fatty acids as the main lipids in the membrane where they contribute to the heat resistance and thermoacidophilic behavior of *Alicyclobacillus* species (Chang and Kang, 2004). Kannenberg et al. (1984) demonstrated that lipids containing ω-cyclohexane fatty acid packed densely which results in low diffusion at high temperatures. The presence of ω-cyclohexyl fatty acids in the cell membrane results in a decrease in the temperature dependence of membrane permeability and a less notable phase transition, which means they stabilize the membrane structure and maintain the resistance against acid and heat stress. The fatty acids also provide protection with the formation of strong hydrophobic bonds which could reduce membrane permeability in extremely acidic and high-temperature condition.

Endospores of *Alicyclobacillus* are also sufficiently heat resistant to enable them to survive in extremely hot and acidic environments. To address endospore formation and its properties, cell cultures at 1 day and 14 days were also observed and showed oval or round-shaped spores in the 14 days culture instead of the long rod-shaped cells seen in the 1 day culture (Supplementary Figure S1). Heat treatment also revealed that the 14 days culture contained 2 to 4 log CFU/mL of heat-resistant endospores, explaining how these strains can thrive in harsh environments. We complemented microscopic evaluation with a genomic analysis of genes in the spore formation pathway and found 50 and 58 sporulation-associated genes in the AL01A and AL05G genome assembly (Supplementary Tables S4 and S5). The identified genes were connected to various aspects such as stage II to V sporulation proteins, RNA polymerase sporulation-specific sigma factor, response regulators within the two-component system, and more.

Antibiotic resistance genes could encode for efflux pumps able to confer resistance against antibiotics and heavy metal toxicity (Aulitto et al., 2021). From the genome information of AL01A and AL05G, the small multidrug resistance antibiotic efflux pump, *vanT*, and glycopeptide resistance gene clusters were detected. The loose hits of the genome sequence also showed a numbers of ABC antibiotic efflux pump, MFS antibiotic efflux pump, and RND antibiotic efflux pump-related genes. The presence of these genes could enable them to cope with heavy metal toxicity since the solubility of heavy metals increases under acidic pH conditions (Knapp et al., 2017; Aulitto et al., 2021). Both AL01A and AL05G genomes also have antibiotic-resistant *fusA* genes conferring resistance to fusidic acid which is effective primarily on bacteria with a Gram-positive type of cell wall. Indeed, the chemical sensitivity test showed that both *Alicyclobacillus* isolates were resistant to fusidic acid.

As AL01A and AL05G were isolated during the study to isolate methanotrophs, central carbohydrate metabolism-related genes were also checked based on the genome information. Most genes related to the intermediary carbon metabolism in the *Alicyclobacillus* isolates exist in the *Methylacidiphilum* genome except the genes encoding phosphoserine aminotransferase (*serC*) and phosphosulfolactate synthase (*comA*). Phosphoserine aminotransferase catalyzes the following chemical reaction:

1. O-phospho-L-serine + 2-oxoglutarate ↔ 3-phosphonooxypyruvate + L-glutamate.
2. 4-phosphonooxy-L-threonine + 2-oxoglutarate ↔ (3R)-3-hydroxy-2-oxo-4-phosphono oxybutanoate + L-glutamate.

which are related to serine and threonine metabolism. Phosphosulfolatate synthase catalyzed the reaction phosphoenolpyruvate + sulfite → (2R)-2-O-phospho-3-sulfolactate. Though AL01A and AL05G genomes do not have genes encoding methanol dehydrogenase (methanol → formaldehyde +2 electrons +2 H^+^), both strains could grow with methanol up to 1.25 mM. Only the AL01A genome also has the gene encoding glycerone kinase (DAK, dihydroxyacetone kinase) catalyzing the glycerone that comes from formaldehyde: ATP + glycerone → ADP + glycerone phosphate. This enzyme transfers phosphorus-containing groups with an alcohol group as an acceptor (Sellinger and Miller, 1957).

While most of the intermediary carbon metabolism-related genes present in the *Alicyclobacillus* isolates also exist in the *Methylacidiphilum* genome, the two organisms utilize different central carbon metabolism pathways. Unlike *Alicyclobacillus* that utilizes glycolysis as one of its central carbon metabolism pathways, *Methylacidiphilum* uses the CBB cycle for producing biomass. Furthermore, we confirmed that *Alicyclobacillus* could grow with methanol and formate, which aligns with the metabolic characteristics of *Methylacidiphilum* (Supplementary Figure S2). These differences in metabolic pathways and growth patterns reflect the unique metabolic adaptations of each organism.

The AL01A and AL05G genomes do not have a nitrate reduction system similar to the other *Alicyclobacillus* strains (Goto et al., 2002). However, it seems that the new isolates could play an important role in sulfur metabolism as both strains have *cysP* encoding sulfate/thiosulfate transport system substrate-binding protein that is responsible for the uptake of extracellular sulfate into the cell (Aguilar-Barajas et al., 2011). The *Alicyclobacillus* isolates then convert sulfate to sulfide through the assimilatory sulfate reduction pathway. Through sulfur metabolism, the bacteria could synthesize organic sulfur compound such as sulfur amino acids and other metabolites, but excess sulfide could also be excreted. Unlike the reference *A. acidocaldarius* genome, however, our genome does not have the genes encoding the sulfonate transport system substrate-binding protein.

Thermoacidophilic bacteria such as *Alicyclobacillus* and *Methylacidiphilum* are adapted to survive in extreme environments with high temperatures and low pH conditions. Therefore, they require specific metabolic pathways to obtain essential nutreints including vitamins (Merino et al., 2019). Hotsprings are an oligotrophic habitat where nutrients are limited and bacteria are exposed to high levels of environmental stress, which can lead to vitamin depletion. Additionally, according to the “Black Queen” hypothesis that proposes that when community members can access essential nutrients and growth factors from their surroundings, they gradually lose their capability to perform functions producing these compounds like vitamins (Culp and Goodman, 2023). These vitamin auxotrophs could arise to reduce the metabolic burden required to produce essential metabolites that have long biosynthetic pathways. Therefore, it is important to understand vitamin auxotrophy between species and strains to identify their interactions. In a previous study, Eloe-Fadrosh et al. (2016) also documented the potential vitamin exchange occurring in microbial communities in hot springs. Here, vitamin metabolism in *Alicyclobacillus* and *Methylacidiphilum* showed that there is a potential syntrophic growth relationship between these two bacterial strains based on vitamin crossfeeding. Genomic analysis shows that they employ different strategies to produce vitamins, where they could potentially complement each other’s deficiencies, thereby resulting in a mutually beneficial growth relationship. Nonetheless, in future studies, it remains crucial to further validate vitamin auxotrophies using viable strains.

Thiamin (vitamin B_1_) is an essential cofactor for all organisms. In thiamin biosynthesis, *Alicyclobacillus* utilizes cysteine and glycine, while *Methylacidiphilum* employs tyrosine in the metabolic pathways. This is similar with *Bacillus subtilis* that utilizes glycine instead of tyrosine to form dehydroglycine unlike *E. coli* (Du et al., 2011). In addition, only *Alicyclobacillus* possess the gene encoding thiamin phosphate phosphatase (*engC*) that is responsible for thiamin production, which suggests that *Methylacidiphilum* may rely on *Alicyclobacillus* for its thiamin needs. The thiamin produced can be transformed into 5-2(-Hydroxyethyl)-4-methylthiazole and 5-2(-Hydroxyethyl)-4-methylthiazole phosphate through the genes in *Alicyclobacillus* genome (*tenA* and *thiM*). It forms thiamin monophosphate mediated by thiamin phosphate synthase (ThiE) and thiamin phosphate kinase (ThiL) then catalyze a phosphorylation step to yield thiamin diphosphate which is an active form of thiamine (Du et al., 2011). Similarly, while both strains have the genes for producing nicotinate (*pncB*), only *Alicyclobacillus* genomes have the genes responsible for mediating intermediate production (*punA, ushA,* and *SIR2*) for nicotinate (vitamin B_3_) metabolism. This suggests that *Methylacidiphilum* may depend on *Alicyclobacillus* for its nicotinate needs, nicotinate is then converted into NAD^+^ through nicotinate phosphoribosyltransferase and NAD synthetase. The produced NAD^+^ acts as a cofactor for enzymes involved in cellular energy metabolism and various cellular functions including metabolic pathways, DNA repair, cellular senescence, and immune function (Amjad et al., 2021; Covarrubias et al., 2021).

Understanding of biotin (vitamin B_7_) biosynthesis is still limited; however, there are two stages proposed by the previous studies: synthesis of a pimelate moiety and the assembly of the bicyclic rings of the biotin molecule (Cronan, 2018; Manandhar and Cronan, 2018). The latter is mostly conserved with four associated genes including *bioF, bioA, bioD*, and *bioB* (Galisteo Gómez et al., 2023). Interestingly, both *Alicyclobacillus* and *Methylacidiphilum* possess most of the *bio* genes involved in biotin production. However, only *Alicyclobacillus* has the *bioF* gene, which connects the pathway for pimeloyl-[acp] or pimeloyl-CoA to 8-amino-7-oxononanoate, which is essential for biotin production. Regarding vitamin K metabolism, the two *Alicyclobacillus* strains utilize different pathways for menaquinone production in contrast to *Methylacidiphilum*. This implies that *Alicyclobacillus* and *Methylacidiphilum* may not rely on each other for this vitamin. Despite the complementary relationship in vitamin metabolisms, further research is required to fully understand the nature of their vitamin auxotrophy.

In summary, we identified and characterized two strains of thermoacidophilic bacteria, *Alicyclobacillus* AL01A and AL05G, from a geothermal environment in Yellowstone National Park. The isolates can grow at low pH and high temperature (optimally at pH 3.5 and 55°C). The presence of ω-alicyclic fatty acids in the membrane (around 60% of the membrane lipids) and sporulation ability contribute to the high thermal resistance of the bacteria. Genome mining performed to target genes involved in antibiotic resistance revealed many multidrug efflux systems, which suggests its ability to tolerate antibiotic and heavy metal toxicity. When comparing with *Methylacidiphilum* sp. YNP IV (GenBank GCA_021323575.1), *Alicyclobacillus* utilize glycolysis as one of its central carbon metabolism pathways, while *Methylacidiphilum* uses the CBB cylcle for producing biomass. AL01A and AL05G genomes also contain the genes that are lacking in the methanotroph genome in the intermediary carbon metabolism. While *Alicyclobacillus* isolates have no genes for nitrite reduction, they could transport extracellular sulfate and metabolize it. In vitamin metabolism, *Alicyclobacillus* and the Verrucomicrobial methanotroph have incomplete pathways, leading to a potentially beneficial relationship, especially in the metabolism of vitamin B_1_, B_3_, and B_7_. Taken together, these genotypic and phenotypical characteristics provide insights into the potential symbiotic role of the new *Alicyclobacillus* isolates with other bacteria in the geothermal environment.

## Data availability statement

The datasets presented in this study can be found in online repositories. The names of the repository/repositories and accession number(s) can be found below: NCBI - PRJNA978622.

## Author contributions

DP, PL, CR, and RM: conceptualization. HK, NK, CR, and RM: methodology and formal analysis. HK: data curation. AP, DP, PL, and RW: resources. HK and RM: writing—original draft preparation. HK, CR, and RM: writing—review and editing. DP, PL, CR, and RM: project administration and funding acquisition. All authors contributed to the article and approved the submitted version.

## Funding

This study was supported by Shell Exploration and Production Inc. (C1610) through the Energy and Biosciencers Institute at the University of California-Berkeley. The funding for this research was provided by Arm & Hammer, and the funder had a role in the planning, execution, and review of the study.

## Conflict of interest

DP and PL are employed by Shell.

The remaining authors declare that the research was conducted in the absence of any commercial or financial relationships that could be construed as a potential conflict of interest.

The handling editor DM-D declared a shared affiliation with the authors HK, NK, AP, RW, CR, and RM at the time of review.

## Publisher’s note

All claims expressed in this article are solely those of the authors and do not necessarily represent those of their affiliated organizations, or those of the publisher, the editors and the reviewers. Any product that may be evaluated in this article, or claim that may be made by its manufacturer, is not guaranteed or endorsed by the publisher.

## Footnotes

^1^https://card.mcmaster.ca/analyze/rgi
