# Supplementary material for: Genomic insight and physiological characterization of thermoacidophilic Alicyclobacillus isolated from Yellowstone National Park
Source: Front Microbiol. 2023 Sep 26;14:1232587. doi: 10.3389/fmicb.2023.1232587 (PMC10562698; doi:10.3389/fmicb.2023.1232587)
Supplement: Supplementary file 1 [file Data_Sheet_1.pdf]

Supplementary information

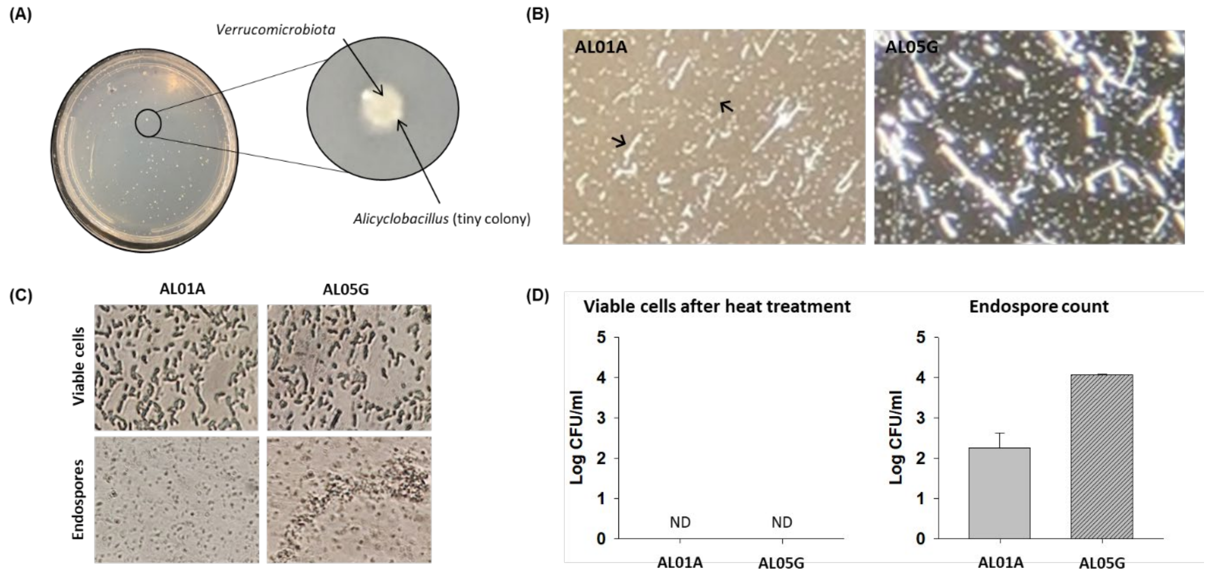

Figure S1. Characteristics of *Alicyclobacillus* isolates. (A) Verrucomicrobiota and *Alicyclobacillus* colonies on the plate. (B) Phase contrast microscopy of AL01A and AL05G (1,000× magnification). (C) Viable cells and endospores of AL01A and AL05G staining with malachite green (5%) and safranin (0.5%) for 1 min. (D) Endospore count of 1 day-culture (viable cells) and 14 day-culture after heat treatment for 10 min at 80ºC.

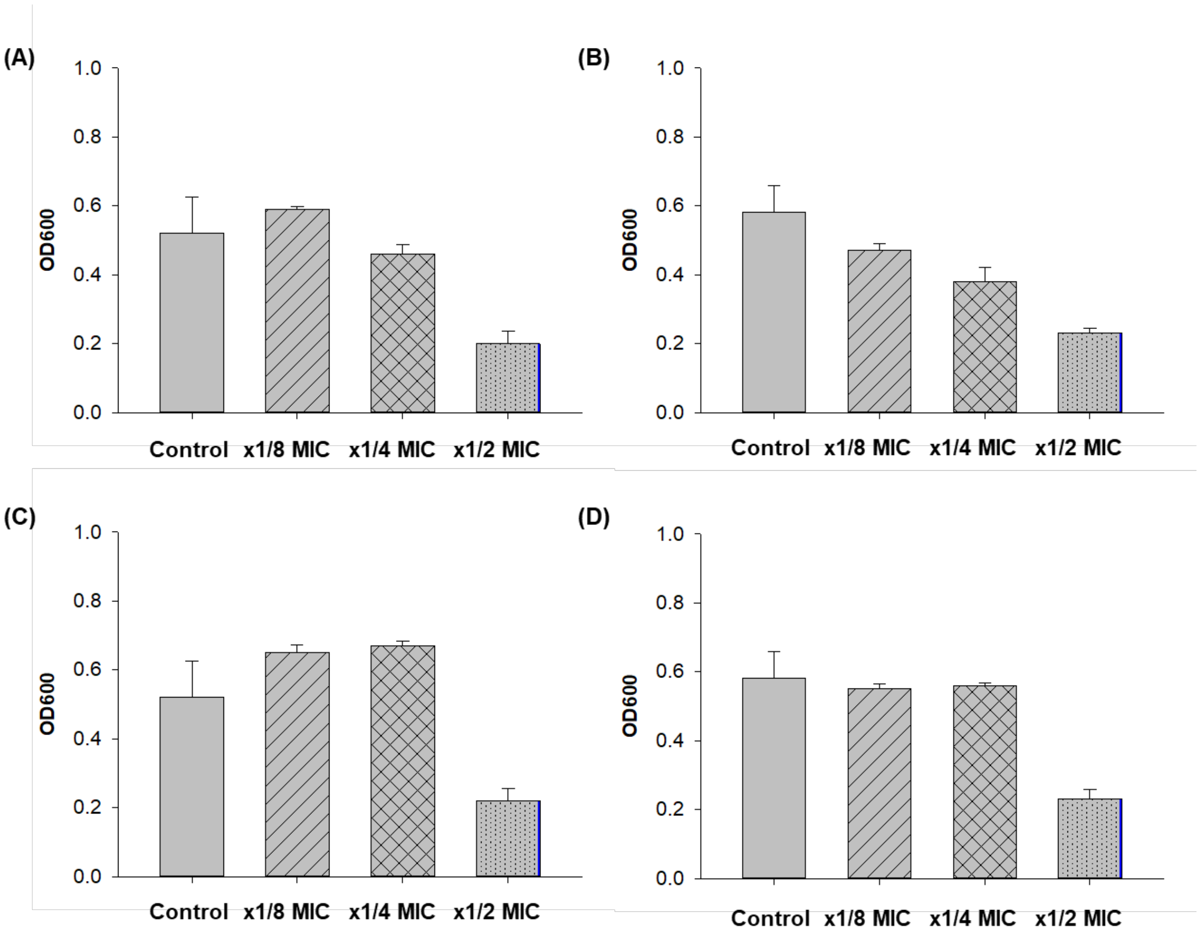

Figure S2. Growth test of (A and C) AL01A and (B and D) AL05G in x1/8, x1/4, and x1/2 MIC of (A and B) methanol and (C and D) formate.

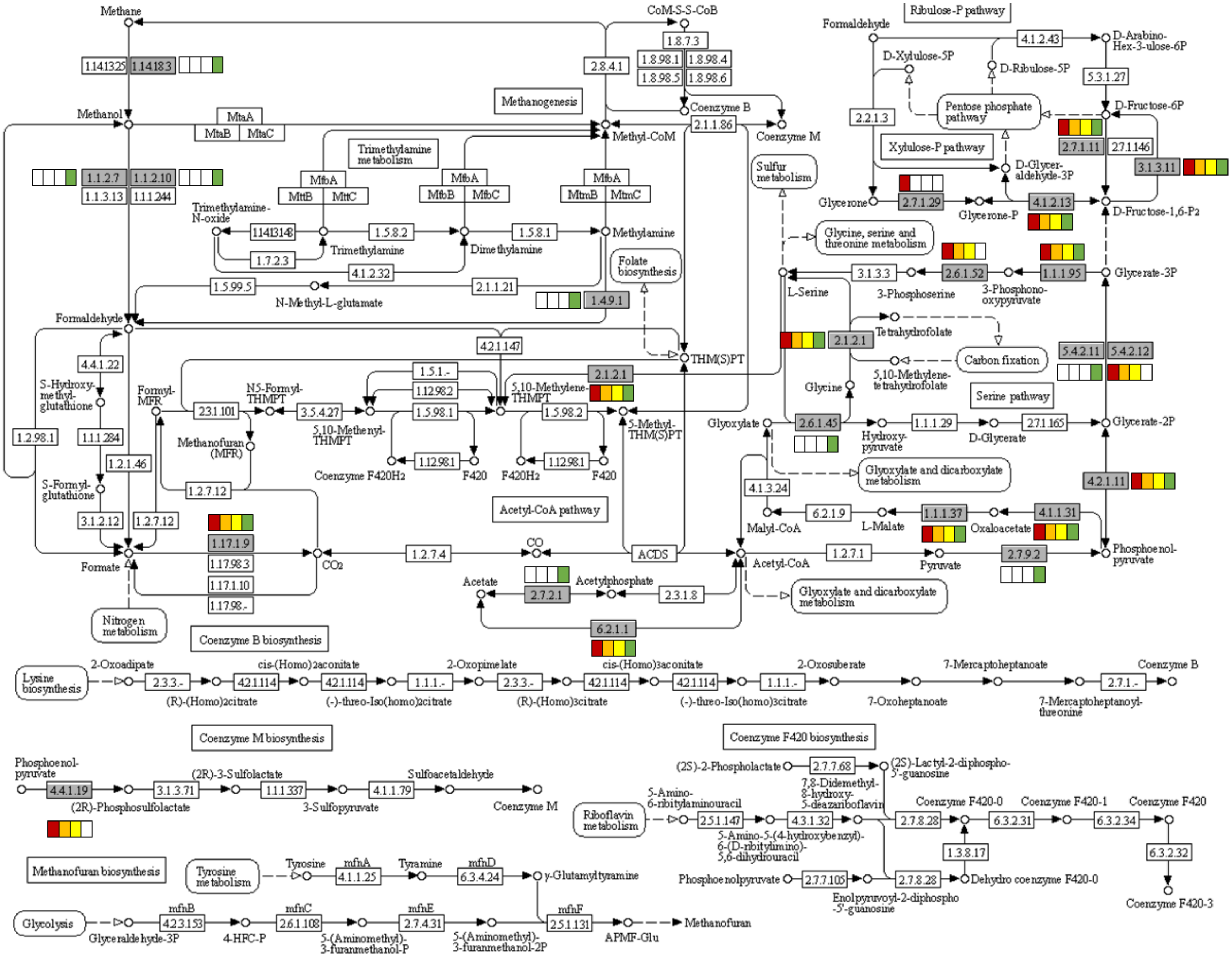

Methane
CoM-S-S-CoB
Ribulose-P pathway
Formaldehyde
1.8.7.3
1.8.98.1
1.8.98.4
1.8.98.5
1.8.98.6
4.1.2.43
D-Arabino-Hex-3-ulose-6P
D-Xylulose-5P
D-Ribulose-5P
5.3.1.27
1.14.13.25
1.14.18.3
2.8.4.1
Methanogenesis
Coenzyme B
D-Fructose-6P
Methanol
MtaA
MtaB
MtaC
2.1.1.86
Methyl-CoM
Coenzyme M
2.2.1.3
Pentose phosphate pathway
Xylulose-P pathway
2.7.1.11
2.7.1.146
3.1.3.11
Trimethylamine metabolism
Sulfur metabolism
D-Glyceraldehyde-3P
1.1.2.7
1.1.2.10
1.1.3.13
1.1.1.244
MtbA
MttB
MttC
MtbA
MtbB
MtbC
MtbA
MtmB
MtmC
Glycerone
2.7.1.29
Glycerone-P
4.1.2.13
D-Fructose-1,6-P2
Trimethylamine-N-oxide
1.14.13.148
1.5.8.2
1.5.8.1
Methylamine
Trimethylamine
Dimethylamine
Glycine, serine and threonine metabolism
1.7.2.3
4.1.2.32
Folate biosynthesis
3.1.3.3
2.6.1.52
1.1.1.95
Glycerate-3P
1.5.99.5
2.1.1.21
L-Serine
3-Phosphoserine
3-Phosphonooxypyruvate
N-Methyl-L-glutamate
1.4.9.1
Formaldehyde
2.1.2.1
Tetrahydrofolate
4.2.1.147
THM(S)PT
Carbon fixation
5.4.2.11
5.4.2.12
4.4.1.22
S-Hydroxymethylglutathione
Formyl-MFR
N5-Formyl-THMPT
1.5.1.-
1.12.98.2
2.1.2.1
5,10-Methylene-THMPT
5,10-Methylenetetrahydrofolate
Glycine
Serine pathway
2.3.1.101
3.5.4.27
1.5.98.1
1.5.98.2
Glyoxylate
2.6.1.45
1.1.1.29
2.7.1.165
Glycerate-2P
1.2.98.1
1.1.1.284
5-Methyl-THM(S)PT
Hydroxypyruvate
D-Glycerate
Methanofuran (MFR)
5,10-Methenyl-THMPT
1.2.1.46
1.12.98.1
1.12.98.1
Glyoxylate and dicarboxylate metabolism
Coenzyme F420H2
F420
F420H2
F420
S-Formylglutathione
1.2.7.12
4.1.3.24
4.2.1.11
Acetyl-CoA pathway
6.2.1.9
1.1.1.37
4.1.1.31
Malyl-CoA
L-Malate
Oxaloacetate
3.1.2.12
1.2.7.12
1.17.1.9
1.2.7.4
CO
ACDS
1.2.7.1
2.7.9.2
Phosphoenolpyruvate
Formate
1.17.98.3
CO2
Acetyl-CoA
Pyruvate
1.17.1.10
Acetate
Acetylphosphate
1.17.98.-
2.7.2.1
2.3.1.8
Glyoxylate and dicarboxylate metabolism
Nitrogen metabolism
6.2.1.1
Coenzyme B biosynthesis
Lysine biosynthesis
2-Oxoadipate
cis-(Homo)2aconitate
2-Oxopimelate
cis-(Homo)3aconitate
2-Oxosuberate
7-Mercaptoheptanoate
Coenzyme B
2.3.3.-
4.2.1.114
4.2.1.114
1.1.1.-
2.3.3.-
4.2.1.114
4.2.1.114
1.1.1.-
2.7.1.-
(R)-(Homo)2citrate
(-)-threo-Iso(homo)2citrate
(R)-(Homo)3citrate
(-)-threo-Iso(homo)3citrate
7-Oxoheptanoate
7-Mercaptoheptanoylthreonine
Coenzyme M biosynthesis
Coenzyme F420 biosynthesis
Phosphoenolpyruvate
(2R)-3-Sulfolactate
Sulfoacetaldehyde
(2S)-2-Phospholactate
2.7.7.68
(2S)-Lactyl-2-diphospho-5'-guanosine
4.4.1.19
3.1.3.71
1.1.1.337
4.1.1.79
7,8-Didemethyl-8-hydroxy-5-deazariboflavin
(2R)-Phosphosulfolactate
3-Sulfopyruvate
Coenzyme M
5-Amino-6-ribitylaminouracil
Coenzyme F420-0
Coenzyme F420-1
Coenzyme F420
Riboflavin metabolism
2.5.1.147
4.3.1.32
2.7.8.28
6.3.2.31
6.3.2.34
5-Amino-5-(4-hydroxybenzyl)-6-(D-ribitylimino)-5,6-dihydrouracil
1.3.8.17
6.3.2.32
Methanofuran biosynthesis
Tyrosine metabolism
Tyrosine
mfnA
Tyramine
mfnD
4.1.1.25
6.3.4.24
γ-Glutamyltyramine
Phosphoenolpyruvate
2.7.7.105
2.7.8.28
Dehydro coenzyme F420-0
Enolpyruvoyl-2-diphospho-5'-guanosine
Coenzyme F420-3
Glycolysis
mfnB
mfnC
mfnE
mfnF
4.2.3.153
2.6.1.108
2.7.4.31
2.5.1.131
Methanofuran
Glyceraldehyde-3P
4-HFC-P
5-(Aminomethyl)-3-furanmethanol-P
5-(Aminomethyl)-3-furanmethanol-2P
APMF-Glu

Figure S3. Presence of genes associated with methane metabolism. Red, orange, yellow, and green indicate the presence of genes in the AL01A, AL05G, *A. acidocaldarius* DSM 446, and *Methylacidiphylum* YNP IV genome. White indicates the absence of genes. Metabolic pathways are adopted from the KEGG metabolic pathway.

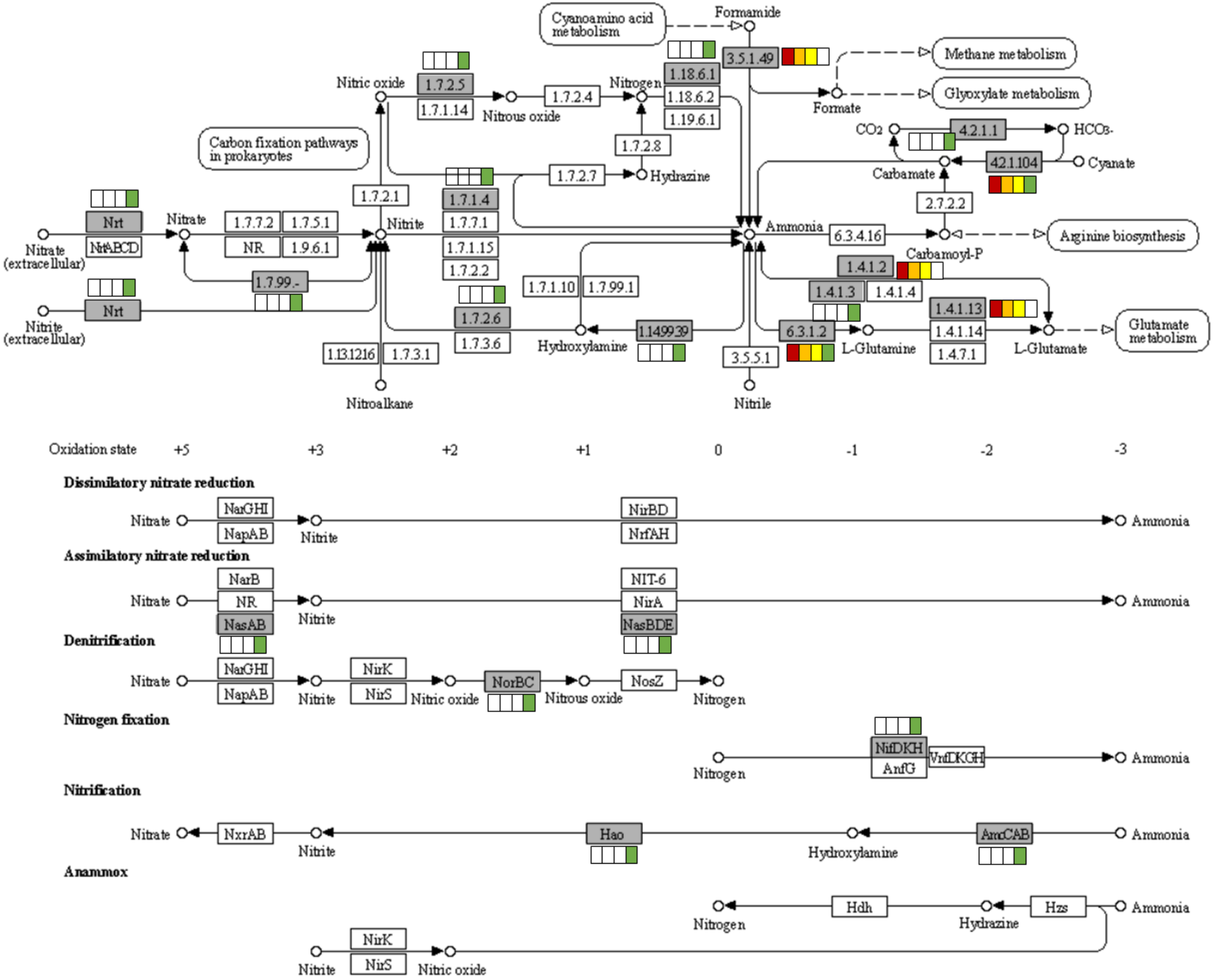

Cyanoamino acid metabolism
Formamide
3.5.1.49
Methane metabolism
Glyoxylate metabolism
Formate
Nitric oxide
1.7.2.5
1.7.1.14
Nitrous oxide
1.7.2.4
Nitrogen
1.18.6.1
1.18.6.2
1.19.6.1
CO2
4.2.1.1
HCO3-
Carbamate
4.2.1.104
Cyanate
Carbon fixation pathways in prokaryotes
1.7.2.8
1.7.2.7
Hydrazine
1.7.2.1
1.7.1.4
1.7.1.1
1.7.1.15
1.7.2.2
2.7.2.2
Nrt
NrtABCD
Nitrate (extracellular)
Nitrate
1.7.7.2
1.7.5.1
NR
1.9.6.1
Nitrite
Ammonia
6.3.4.16
Arginine biosynthesis
Carbamoyl-P
1.4.1.2
1.4.1.3
1.4.1.4
1.7.99.-
Nrt
Nitrite (extracellular)
1.7.1.10
1.7.99.1
1.4.1.13
1.7.2.6
1.14.99.39
6.3.1.2
L-Glutamine
1.4.1.14
L-Glutamate
Glutamate metabolism
1.13.12.16
1.7.3.1
1.7.3.6
Hydroxylamine
3.5.5.1
1.4.7.1
Nitroalkane
Nitrile
Oxidation state
+5
+3
+2
+1
0
-1
-2
-3
Dissimilatory nitrate reduction
Nitrate
NarGHI
NapAB
Nitrite
NirBD
NrfAH
Ammonia
Assimilatory nitrate reduction
Nitrate
NarB
NR
NasAB
Nitrite
NIT-6
NirA
NasBDE
Ammonia
Denitrification
Nitrate
NarGHI
NapAB
Nitrite
NirK
NirS
Nitric oxide
NorBC
Nitrous oxide
NosZ
Nitrogen
Nitrogen fixation
Nitrogen
NifDKH
AnfG
VnfDKGH
Ammonia
Nitrification
Nitrate
NxrAB
Nitrite
Hao
Hydroxylamine
AmoCAB
Ammonia
Anammox
Nitrogen
Hdh
Hydrazine
Hzs
Ammonia
Nitrite
NirK
NirS
Nitric oxide

Figure S4. Presence of genes associated with nitrogen metabolism. Red, orange, yellow, and green indicate the presence of genes in the AL01A, AL05G, *A. acidocaldarius* DSM 446, and *Methylacidiphylum* YNP IV genome. White indicates the absence of genes. Metabolic pathways are adopted from the KEGG metabolic pathway.

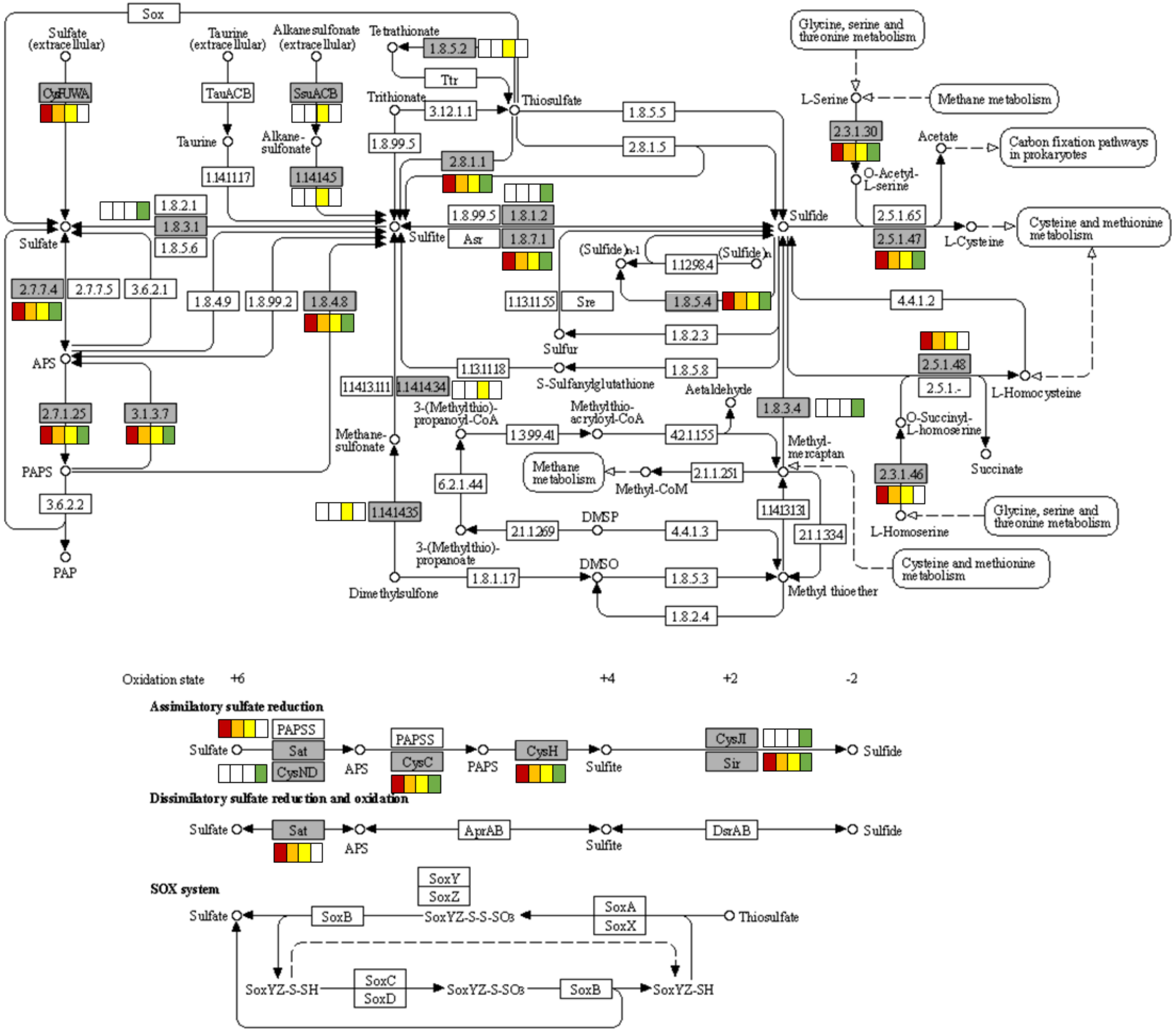

Figure S5. Presence of genes associated with sulfur metabolism. Red, orange, yellow, and green indicate the presence of genes in the

AL01A, AL05G, *A. acidocaldarius* DSM 446, and *Methylacidphylum* YNP IV genome. White indicates the absence of genes. Metabolic pathways are adopted from the KEGG metabolic pathway.

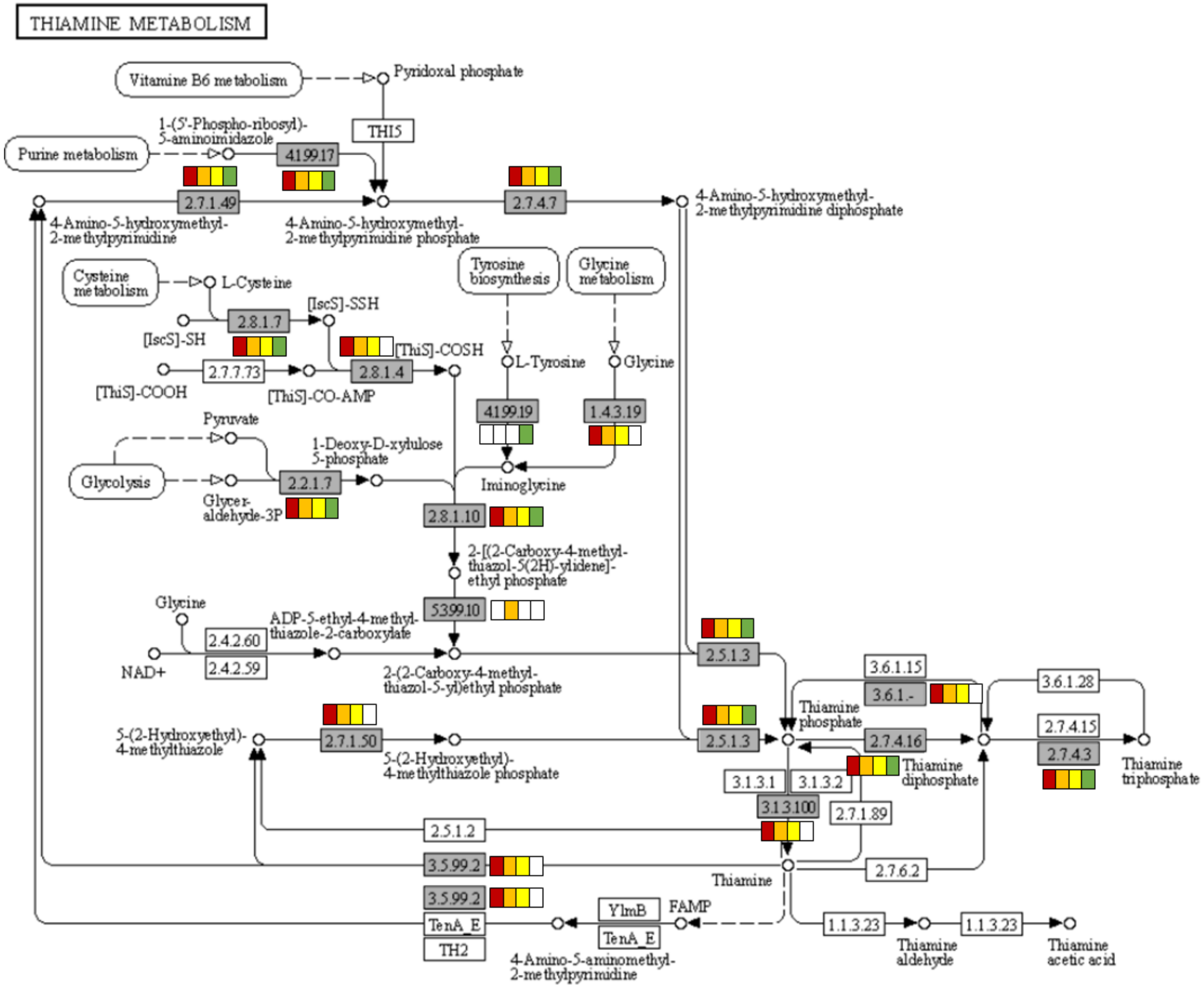
THIAMINE METABOLISM
Vitamine B6 metabolism
Pyridoxal phosphate
THI5
Purine metabolism
1-(5'-Phospho-ribosyl)-5-aminoimidazole
4.1.99.17
2.7.1.49
2.7.4.7
4-Amino-5-hydroxymethyl-2-methylpyrimidine
4-Amino-5-hydroxymethyl-2-methylpyrimidine phosphate
4-Amino-5-hydroxymethyl-2-methylpyrimidine diphosphate
Cysteine metabolism
L-Cysteine
[IscS]-SSH
[IscS]-SH
2.8.1.7
[ThiS]-COSH
[ThiS]-COOH
2.7.7.73
2.8.1.4
[ThiS]-CO-AMP
Tyrosine biosynthesis
Glycine metabolism
L-Tyrosine
Glycine
4.1.99.19
1.4.3.19
Iminoglycine
Pyruvate
Glycolysis
1-Deoxy-D-xylulose 5-phosphate
2.2.1.7
Glyceraldehyde-3P
2.8.1.10
2-[(2-Carboxy-4-methylthiazol-5(2H)-ylidene]-ethyl phosphate
5.3.99.10
Glycine
ADP-5-ethyl-4-methylthiazole-2-carboxylate
2.4.2.60
2.4.2.59
NAD+
2-(2-Carboxy-4-methylthiazol-5-yl)ethyl phosphate
2.5.1.3
3.6.1.15
3.6.1.-
3.6.1.28
Thiamine phosphate
5-(2-Hydroxyethyl)-4-methylthiazole
2.7.1.50
5-(2-Hydroxyethyl)-4-methylthiazole phosphate
2.5.1.3
2.7.4.16
2.7.4.15
2.7.4.3
Thiamine diphosphate
Thiamine triphosphate
3.1.3.1
3.1.3.2
3.1.3.100
2.7.1.89
2.5.1.2
3.5.99.2
Thiamine
2.7.6.2
3.5.99.2
YlmB
FAMP
TenA_E
TenA_E
TH2
4-Amino-5-aminomethyl-2-methylpyrimidine
1.1.3.23
1.1.3.23
Thiamine aldehyde
Thiamine acetic acid

Figure S6. Presence of genes associated with thiamine metabolism. Red, orange, yellow, and green indicate the presence of genes in the AL01A, AL05G, *A. acidocaldarius* DSM 446, and *Methylacidphylum* YNP IV genome. White indicates the absence of genes. Metabolic pathways are adopted from the KEGG metabolic pathway.

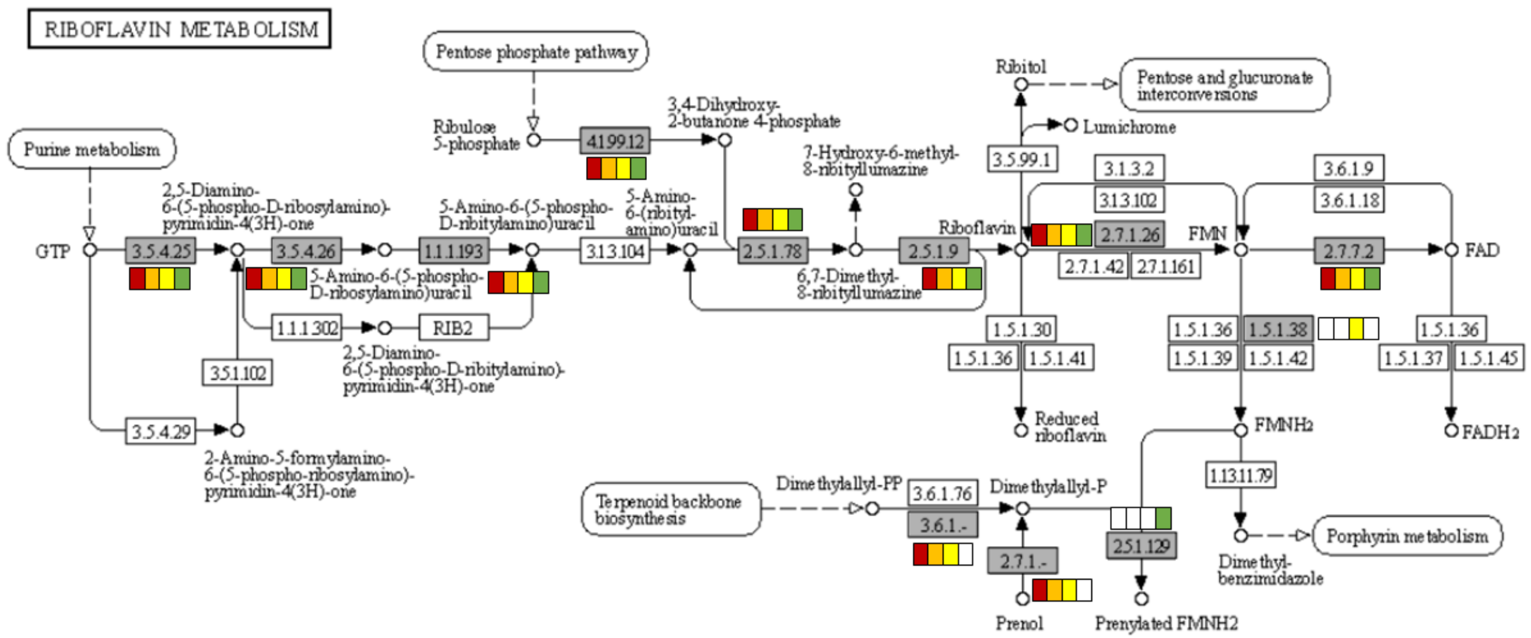

Figure S7. Presence of genes associated with riboflavin metabolism. Red, orange, yellow, and green indicate the presence of genes in the AL01A, AL05G, *A. acidocaldarius* DSM 446, and *Methylacidphylum* YNP IV genome. White indicates the absence of genes. Metabolic pathways are adopted from the KEGG metabolic pathway.

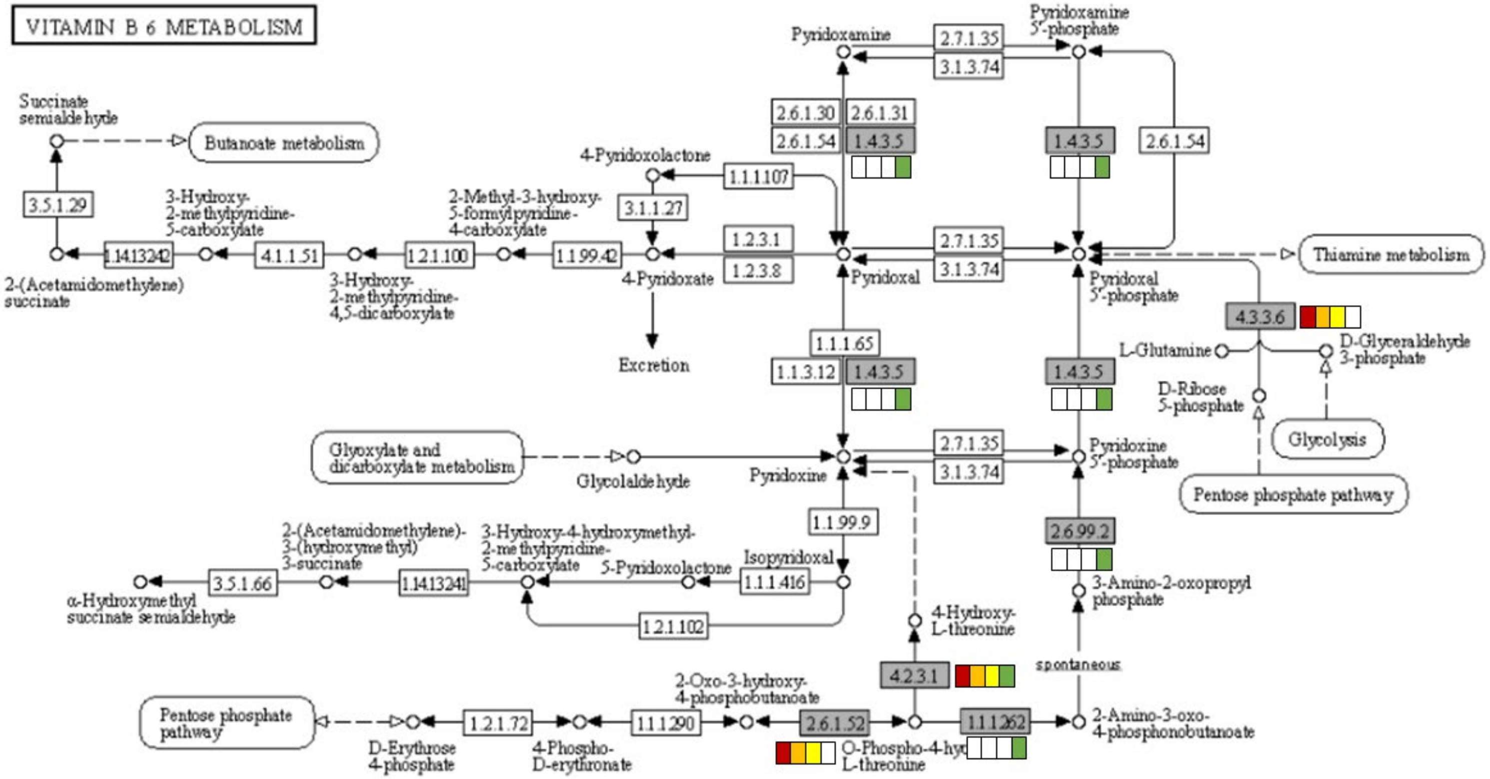

Figure S8. Presence of genes associated with vitamin B_6 metabolism. Red, orange, yellow, and green indicate the presence of genes in the AL01A, AL05G, *A. acidocaldarius* DSM 446, and *Methylacidphylum* YNP IV genome. White indicates the absence of genes. Metabolic pathways are adopted from the KEGG metabolic pathway.

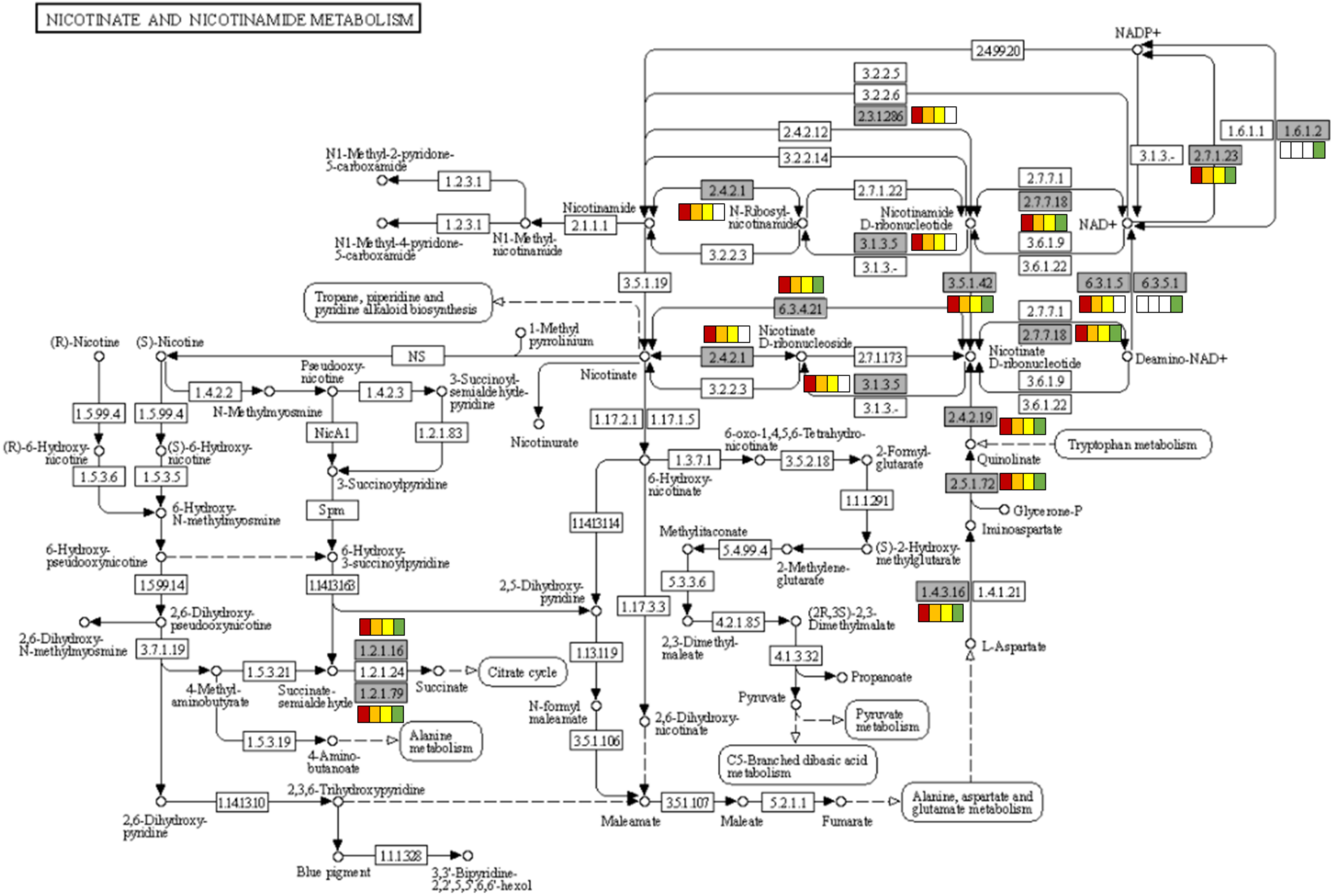

Figure S9. Presence of genes associated with nicotinate and nicotinamide metabolism. Red, orange, yellow, and green indicate the presence

of genes in the AL01A, AL05G, *A. acidocaldarius* DSM 446, and *Methylacidphylum* YNP IV genome. White indicates the absence of genes. Metabolic pathways are adopted from the KEGG metabolic pathway.

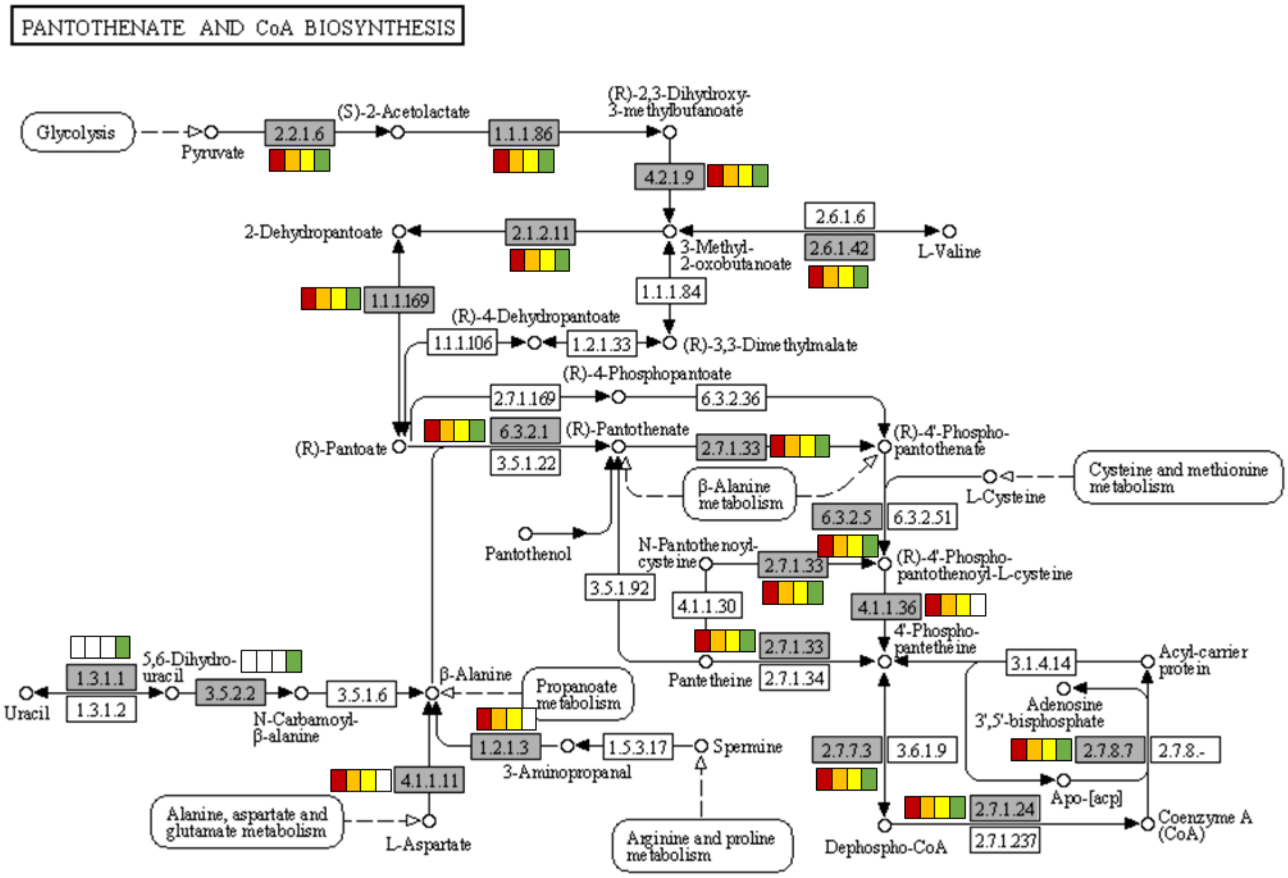

Figure S10. Presence of genes associated with pantothenate and CoA biosynthesis metabolism. Red, orange, yellow, and green indicate the presence of genes in the AL01A, AL05G, *A. acidocaldarius* DSM 446, and *Methylacidphylum* YNP IV genome. White indicates the absence of genes. Metabolic pathways are adopted from the KEGG metabolic pathway.

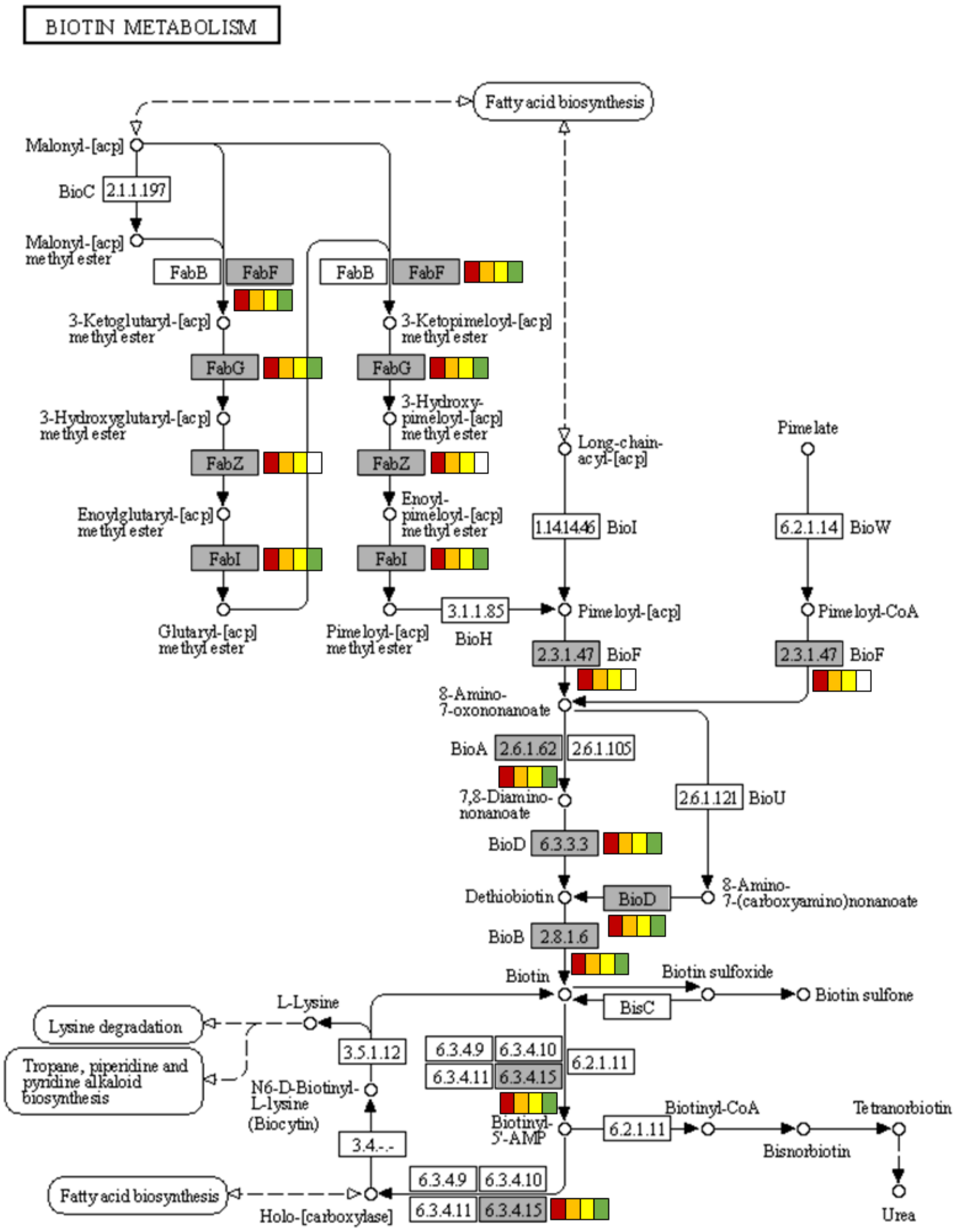

Figure S11. Presence of genes associated with biotin metabolism. Red, orange, yellow, and green indicate the presence of genes in the AL01A, AL05G, *A. acidocaldarius* DSM 446, and *Methylacidphylum* YNP IV genome. White indicates the absence of genes. Metabolic pathways are adopted from the KEGG metabolic pathway.

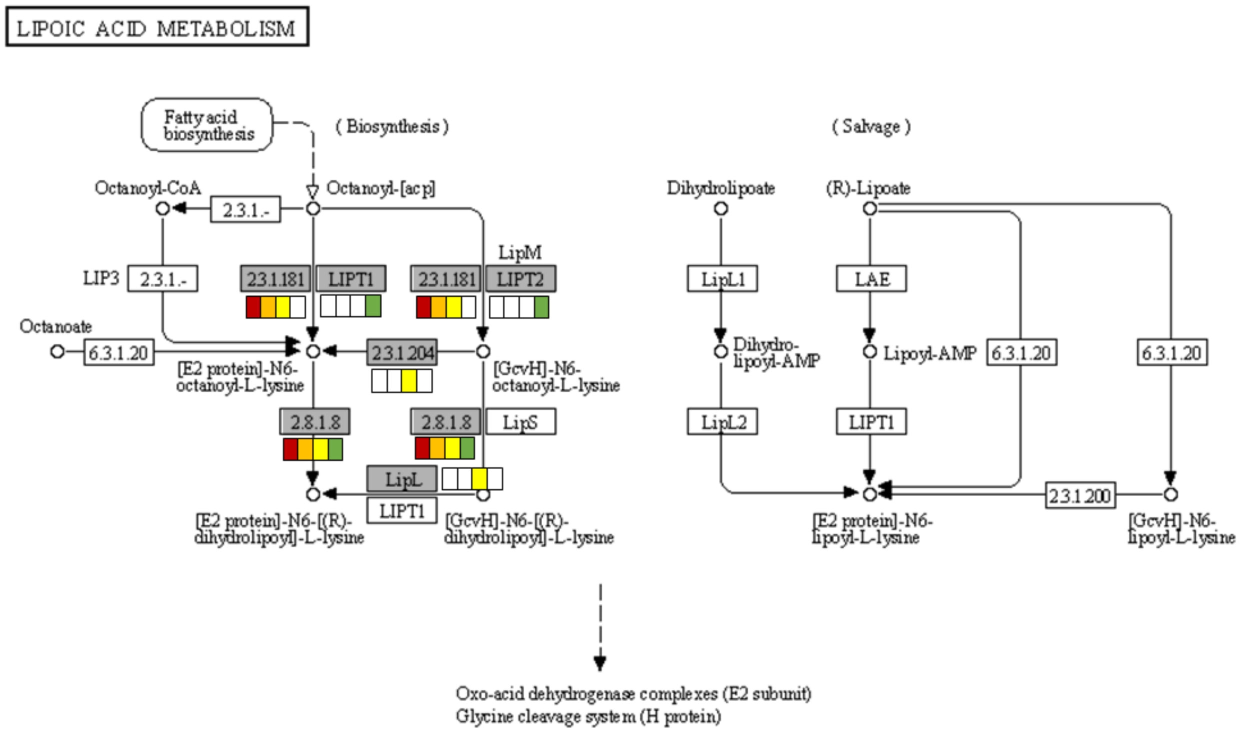

Figure S12. Presence of genes associated with lipoic acid metabolism. Red, orange, yellow, and green indicate the presence of genes in the AL01A, AL05G, *A. acidocaldarius* DSM 446, and *Methylacidphylum* YNP IV genome. White indicates the absence of genes. Metabolic pathways are adopted from the KEGG metabolic pathway.

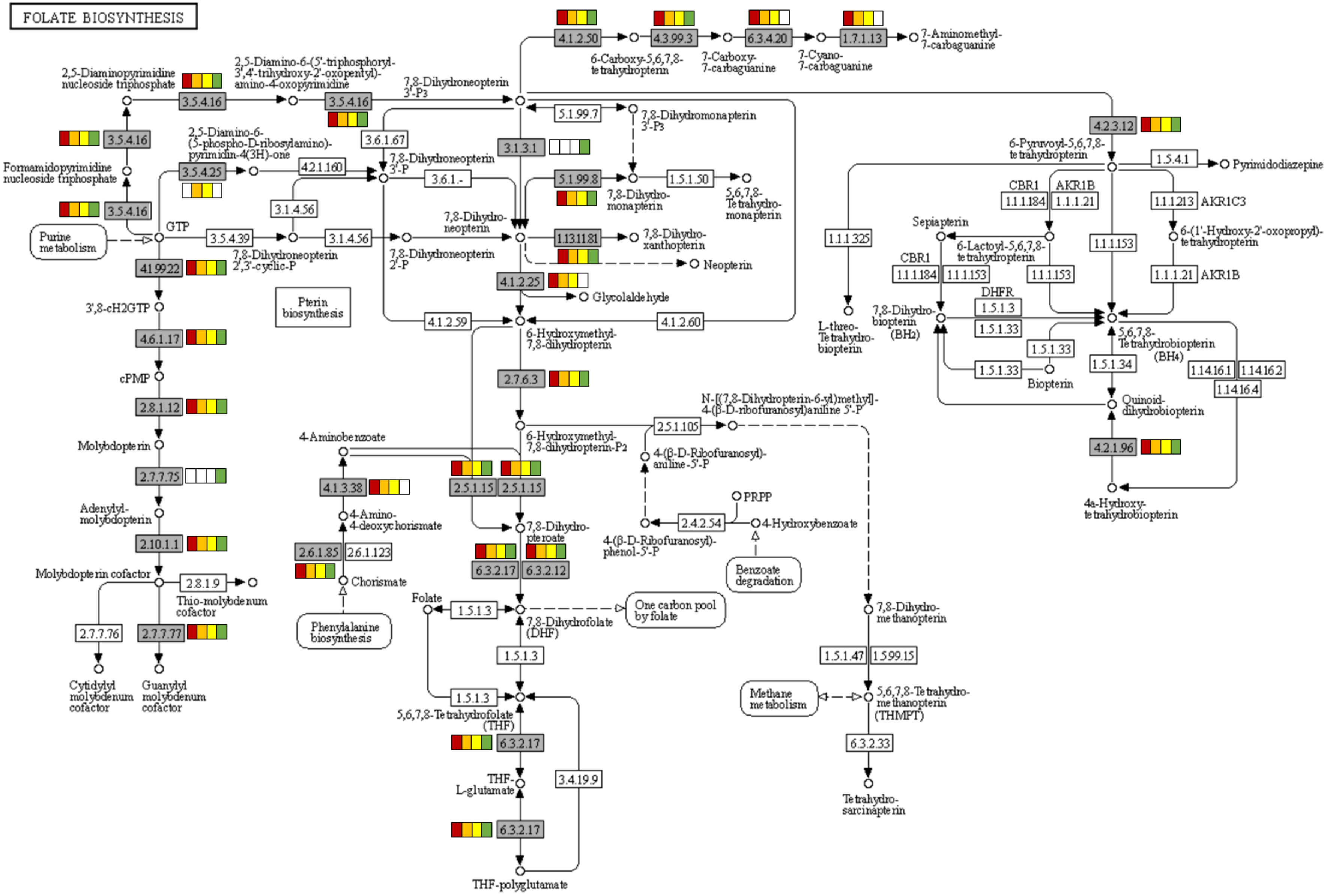
FOLATE BIOSYNTHESIS
2,5-Diaminopyrimidine nucleoside triphosphate
2,5-Diamino-6-(5'-triphosphoryl-3',4'-trihydroxy-2'-oxopentyl)-amino-4-oxopyrimidine
7,8-Dihydroneopterin 3'-P3
3.5.4.16
4.1.2.50
4.3.99.3
6.3.4.20
1.7.1.13
7-Aminomethyl-7-carbaguanine
6-Carboxy-5,6,7,8-tetrahydropterin
7-Carboxy-7-carbaguanine
7-Cyano-7-carbaguanine
5.1.99.7
7,8-Dihydromonapterin 3'-P3
4.2.3.12
6-Pyruvoyl-5,6,7,8-tetrahydropterin
1.5.4.1
Pyrimidodiazepine
Formamidopyrimidine nucleoside triphosphate
2,5-Diamino-6-(5-phospho-D-ribosylamino)-pyrimidin-4(3H)-one
3.5.4.25
4.2.1.160
3.6.1.67
7,8-Dihydroneopterin 3'-P
3.6.1.-
3.1.3.1
5.1.99.8
1.5.1.50
7,8-Dihydromonapterin
5,6,7,8-Tetrahydromonapterin
3.1.4.56
CBR1
AKR1B
1.1.1.184
1.1.1.21
1.1.1.213
AKR1C3
Purine metabolism
GTP
3.5.4.39
7,8-Dihydroneopterin 2',3'-cyclic-P
7,8-Dihydroneopterin 2'-P
7,8-Dihydroneopterin
1.13.11.81
7,8-Dihydroxanthopterin
Neopterin
1.1.1.325
Sepiapterin
6-Lactoyl-5,6,7,8-tetrahydropterin
1.1.1.153
6-(1'-Hydroxy-2'-oxopropyl)-tetrahydropterin
4.1.99.22
4.1.2.25
Glycolaldehyde
3',8-cH2GTP
Pterin biosynthesis
4.1.2.59
4.1.2.60
6-Hydroxymethyl-7,8-dihydropterin
DHFR
1.5.1.3
1.5.1.33
7,8-Dihydrobiopterin (BH2)
L-threo-Tetrahydrobiopterin
5,6,7,8-Tetrahydrobiopterin (BH4)
Biopterin
1.5.1.34
1.14.16.1
1.14.16.2
1.14.16.4
4.6.1.17
cPMP
2.7.6.3
2.8.1.12
Molybdopterin
N-[(7,8-Dihydropterin-6-yl)methyl]-4-(β-D-ribofuranosyl)aniline 5'-P
2.5.1.105
Quinoid-dihydrobiopterin
4-Aminobenzoate
6-Hydroxymethyl-7,8-dihydropterin-P2
4-(β-D-Ribofuranosyl)-aniline-5'-P
4.2.1.96
2.7.7.75
4.1.3.38
2.5.1.15
PRPP
4a-Hydroxy-tetrahydrobiopterin
Adenylyl-molybdopterin
4-Amino-4-deoxychorismate
7,8-Dihydropteroate
2.4.2.54
4-Hydroxybenzoate
4-(β-D-Ribofuranosyl)-phenol-5'-P
2.10.1.1
2.6.1.85
2.6.1.123
6.3.2.17
6.3.2.12
Benzoate degradation
Molybdopterin cofactor
2.8.1.9
Chorismate
Thio-molybdenum cofactor
Folate
One carbon pool by folate
7,8-Dihydro-methanopterin
7,8-Dihydrofolate (DHF)
Phenylalanine biosynthesis
2.7.7.76
2.7.7.77
1.5.1.47
1.5.99.15
Cytidylyl molybdenum cofactor
Guanylyl molybdenum cofactor
Methane metabolism
5,6,7,8-Tetrahydro-methanopterin (THMPT)
5,6,7,8-Tetrahydrofolate (THF)
6.3.2.33
THF-L-glutamate
3.4.19.9
Tetrahydro-sarcinapterin
THF-polyglutamate

Figure S13. Presence of genes associated with folate biosynthesis metabolism. Red, orange, yellow, and green indicate the presence of genes in the AL01A, AL05G, *A. acidocaldarius* DSM 446, and *Methylacidphylum* YNP IV genome. White indicates the absence of genes. Metabolic pathways are adopted from the KEGG metabolic pathway.

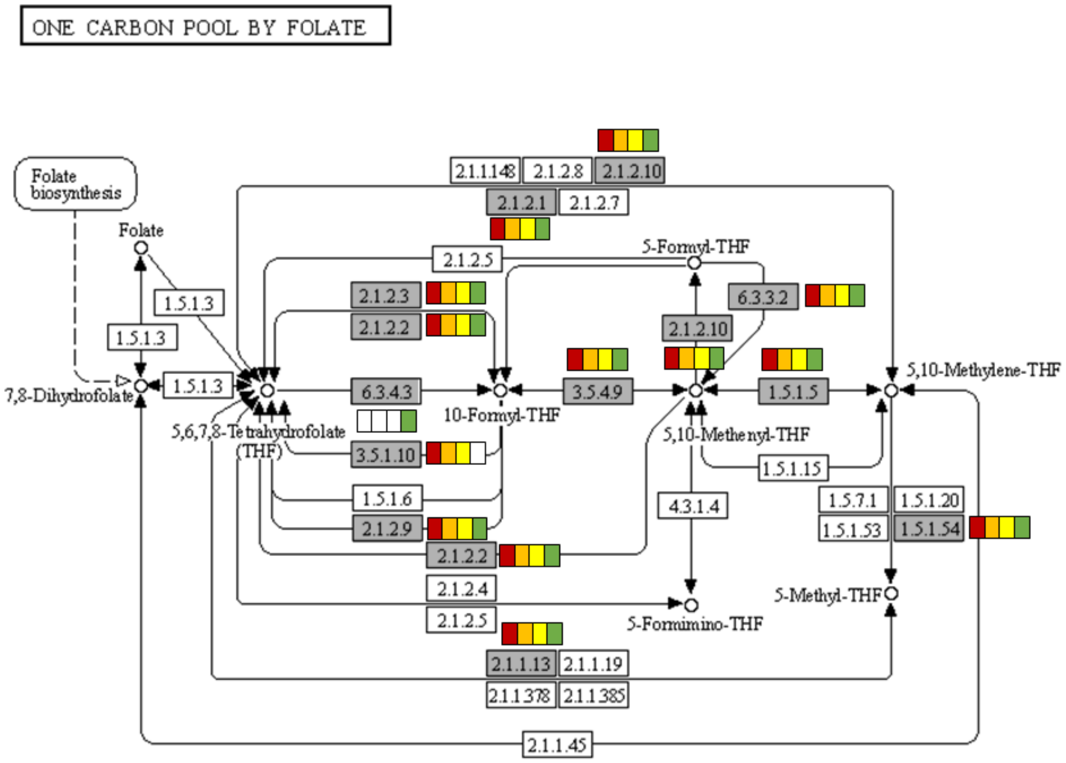

Figure S14. Presence of genes associated with pathways that utilize biotin as a c-factor by folate. Red, orange, yellow, and green indicate the presence of genes in the AL01A, AL05G, *A. acidocaldarius* DSM 446, and *Methylacidphylum* YNP IV genome. White indicates the absence of genes. Metabolic pathways are adopted from the KEGG metabolic pathway.

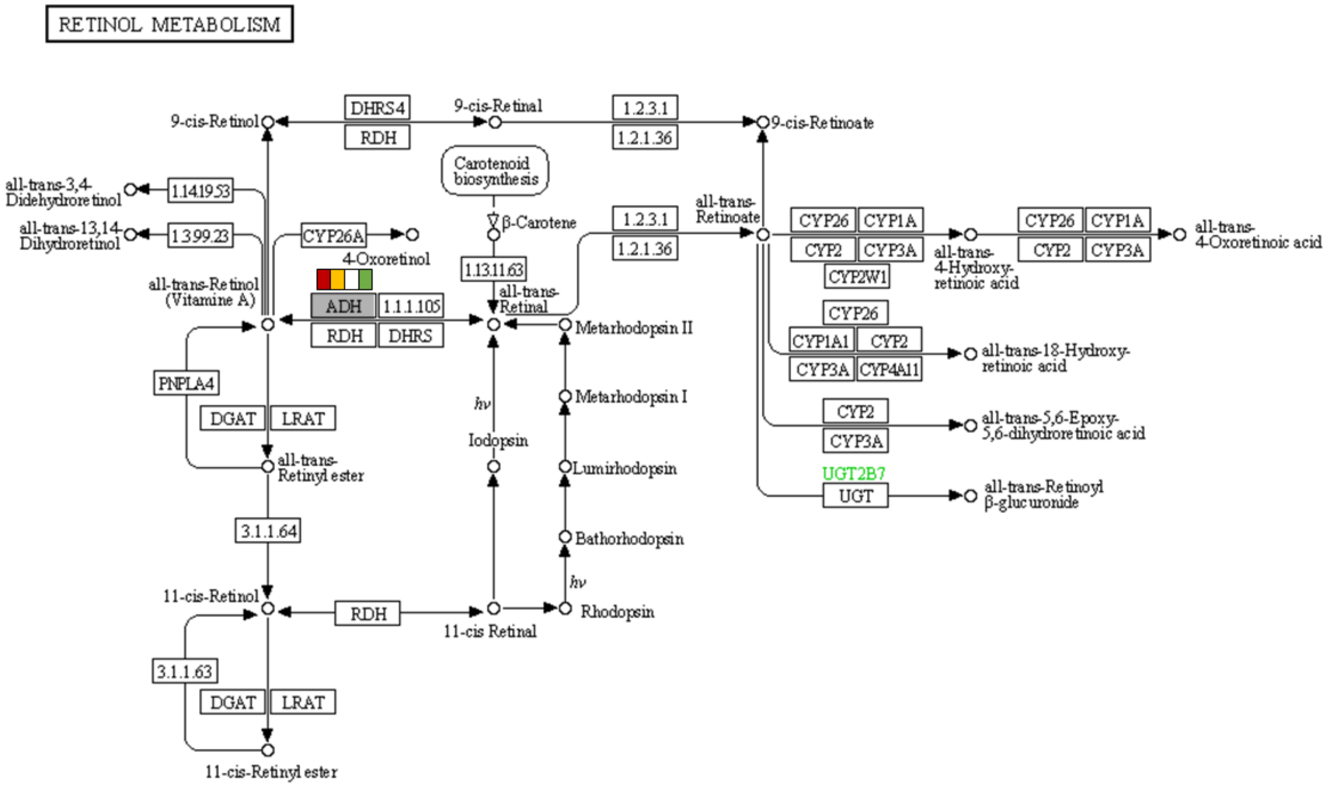

Figure S15. Presence of genes associated with retinol metabolism. Red, orange, yellow, and green indicate the presence of genes in the AL01A, AL05G, *A. acidocaldarius* DSM 446, and *Methylacidphylum* YNP IV genome. White indicates the absence of genes. Metabolic pathways are adopted from the KEGG metabolic pathway.

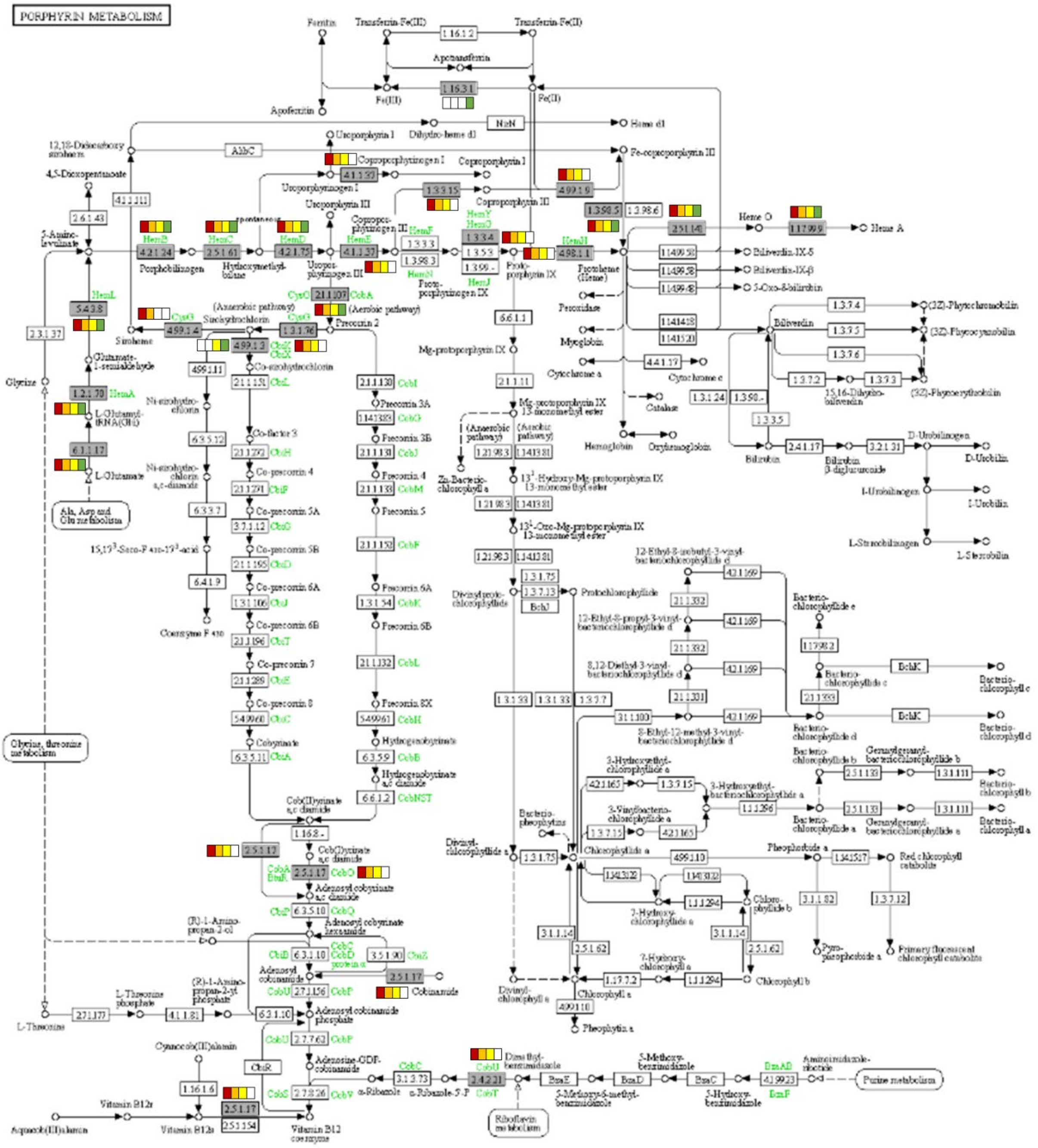

Figure S16. Presence of genes associated with porphyrin metabolism. Red, orange, yellow, and green indicate the presence of genes in the AL01A, AL05G, *A. acidocaldarius* DSM 446, and *Methylacidphylum* YNP IV genome. White indicates the absence of genes. Metabolic pathways are adopted from the KEGG metabolic pathway.

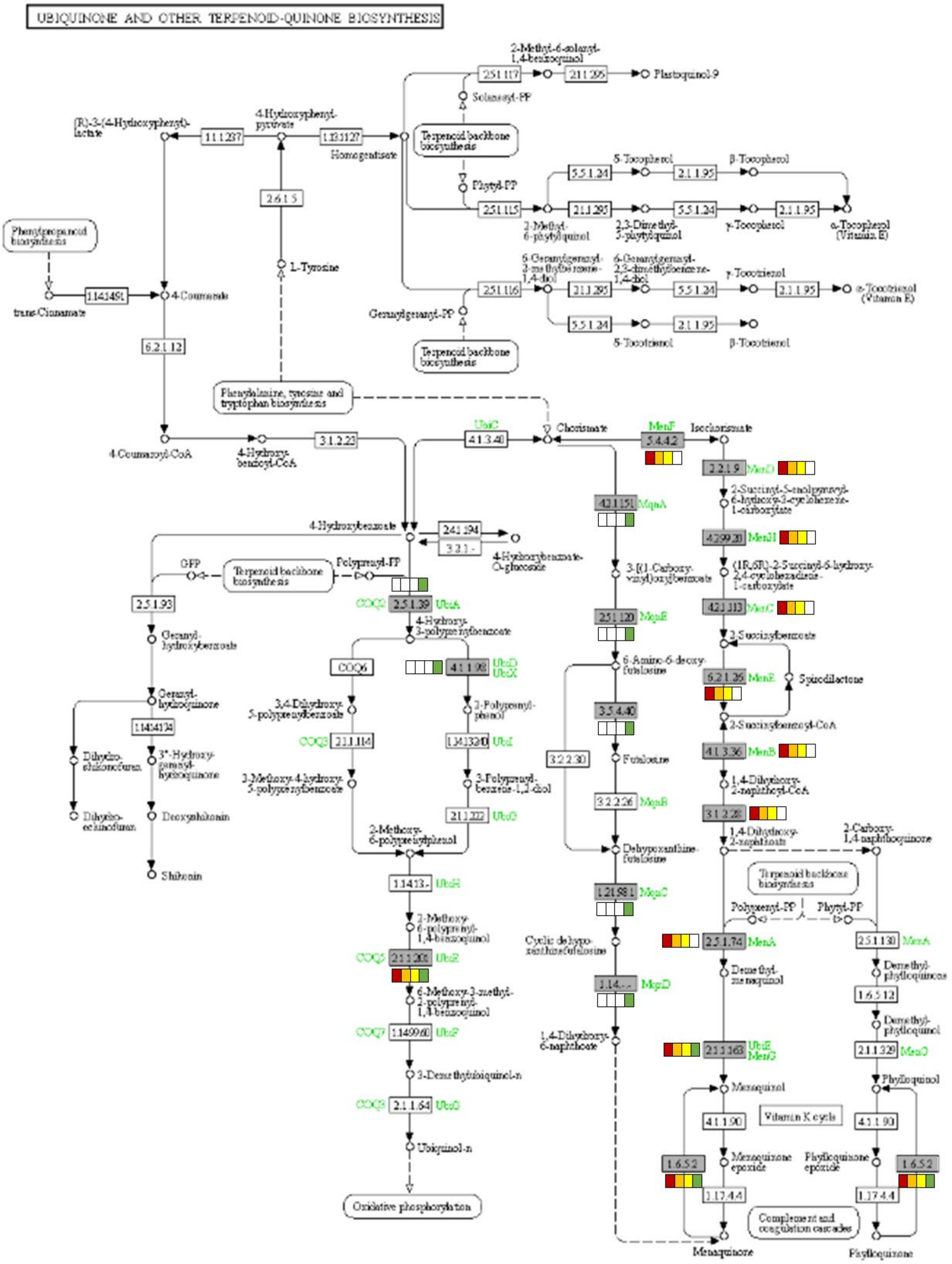

Figure S17. Presence of genes associated with ubiquinone and othert terpenoid-quinone biosynthesis metabolism. Red, orange, yellow, and green indicate the presence of genes in the

AL01A, AL05G, *A. acidocaldarius* DSM 446, and *Methylacidphylum* YNP IV genome. White indicates the absence of genes. Metabolic pathways are adopted from the KEGG metabolic pathway.

Table S1. Composition of mineral medium V42

**Component**	**g/L**
NH_4Cl	0.4
KH_2PO_4	0.05
$MgSO_4 \cdot 7H_2O$	0.02
$CaCl_2 \cdot 6H_2O$	0.01
Yeast extract (0.2 µm filtered)	0.01
$MgCl_2 \cdot 6H_2O$	1
Phytagel	15
**Solution**	**ml/L**
FeEDTA sol.	3 ml
Trace element sol 1	3 ml
Trace element sol 2	1 ml
Vitamin mixture	20 ml

* Adjust pH to 3.5 with sulfuric acid

FeEDTA solution

**Component**	**g/L**
$FeSO_4 \cdot 7H_2O$	1.54
Na_2EDTA	2.06

Trace element sol1

**Component**	**g/L**
$ZnSO_4 \cdot 7H_2O$	0.44
$CuSO_4 \cdot 5H_2O$	0.2
$MnCl_2 \cdot 4H_2O$	0.19
$Na_2MoO_4 \cdot 2H_2O$	0.6
H_3BO_3	0.1
$CoCl_2 \cdot 6H_2O$	0.08

Trace element sol2

**Component**	**g/L**
Nitrilotriacetic acid	1.5
$Fe(NH_4)2(SO_4)_2 \cdot H_2O$	0.2
Na_2SeO_3	0.2

$CoCl_2 \cdot 6H_2O$	0.1
$MnSO_4 \cdot 7H_2O$	0.16
$Na_2MoO_4 \cdot 2H_2O$	0.1
$Na_2WO_4 \cdot 2H_2O$	0.1
$ZnSO_4 \cdot 7H_2O$	0.1
$AlK(SO_4)_2$	0.08
$NiCl_2 \cdot 6H_2O$	0.025
H_3BO_3	0.01
$CuSO_4 \cdot 5H_2O$	0.01

Rare earth elements

**Component**	**µM**
$CeCl_3 \cdot 7H_2O$	0.2
$LaCl_3 \cdot 7H_2O$	0.2

Table S2. Number of antimicrobial resistant (AMR) gene families in the *Alicyclobacillus* AL01A genome assembly

**AMR gene family**	**No.**	**RGI criteria**
Small multidrug resistance (SMR) antibiotic efflux pump	1	Strict
vanT	1	Strict
Glycopeptide resistance gene cluster	1	Strict
ATP-binding cassette (ABC) antibiotic efflux pump	50	Loose
major facilitator superfamily (MFS) antibiotic efflux pump	44	Loose
resistance-nodulation-cell division (RND) antibiotic efflux pump	25	Loose
glycopeptide resistance gene cluster	9	Loose
pmr phosphoethanolamine transferase	9	Loose
Penicillin-binding protein mutations conferring resistance to beta-lactam antibiotics	6	Loose
antibiotic resistant fabI	3	Loose
vanH	3	Loose
vanR	3	Loose
antibiotic-resistant isoleucyl-tRNA synthetase (ileS)	3	Loose
tetracycline-resistant ribosomal protection protein	3	Loose
undecaprenyl pyrophosphate related proteins	3	Loose
antibiotic-resistant murA transferase	2	Loose
elfamycin resistant EF-Tu	2	Loose
vanS	2	Loose
helicase-like RNA polymerase protection protein	2	Loose
methicillin resistant PBP2	2	Loose
daptomycin resistant cls	1	Loose
antibiotic resistant gidB	1	Loose
fluoroquinolone resistant gyrB	1	Loose
fluoroquinolone resistant gyrA	1	Loose
antibiotic resistant fusE	1	Loose
antibiotic resistant ndh	1	Loose
vanT	1	Loose
sulfonamide resistant sul	1	Loose
Erm 23S ribosomal RNA methyltransferase	1	Loose
kdpDE	1	Loose
msr-type ABC-F protein	1	Loose
Outer Membrane Porin (Opr)	1	Loose
Cfr 23S ribosomal RNA methyltransferase	1	Loose
Intrinsic peptide antibiotic resistant Lps	1	Loose
Van ligase	1	Loose
Miscellaneous ABC-F subfamily ATP-binding cassette ribosomal protection proteins	1	Loose
rifamycin-resistant beta-subunit of RNA polymerase (rpoB)	1	Loose
antibiotic resistant fusA	1	Loose

Table S3. Number of antimicrobial resistant (AMR) gene families in the AL05G genome assembly

AMR gene family	No.	RGI criteria
Small multidrug resistance (SMR) antibiotic efflux pump	1	Strict
vanT	1	Strict
Glycopeptide resistance gene cluster	1	Strict
ATP-binding cassette (ABC) antibiotic efflux pump	55	Loose
major facilitator superfamily (MFS) antibiotic efflux pump	45	Loose
resistance-nodulation-cell division (RND) antibiotic efflux pump	26	Loose
pmr phosphoethanolamine transferase	10	Loose
glycopeptide resistance gene cluster	9	Loose
Penicillin-binding protein mutations conferring resistance to beta-lactam antibiotics	6	Loose
antibiotic-resistant murA transferase	4	Loose
vanR	3	Loose
vanH	3	Loose
undecaprenyl pyrophosphate related proteins	3	Loose
tetracycline-resistant ribosomal protection protein	3	Loose
antibiotic-resistant isoleucyl-tRNA synthetase (ileS)	3	Loose
antibiotic resistant fabI	3	Loose
vanS	2	Loose
Van ligase	2	Loose
Miscellaneous ABC-F subfamily ATP-binding cassette ribosomal protection proteins	2	Loose
methicillin resistant PBP2	2	Loose
helicase-like RNA polymerase protection protein	2	Loose
Erm 23S ribosomal RNA methyltransferase	2	Loose
elfamycin resistant EF-Tu	2	Loose
vanT	1	Loose
sulfonamide resistant sul	1	Loose
rifamycin-resistant beta-subunit of RNA polymerase (rpoB)	1	Loose
Outer Membrane Porin (Opr)	1	Loose
msr-type ABC-F protein	1	Loose
kdpDE	1	Loose
Intrinsic peptide antibiotic resistant Lps	1	Loose
fluoroquinolone resistant gyrB	1	Loose
fluoroquinolone resistant gyrA	1	Loose
daptomycin resistant cls	1	Loose
Cfr 23S ribosomal RNA methyltransferase	1	Loose
antibiotic resistant ndh	1	Loose
antibiotic resistant gidB	1	Loose
antibiotic resistant fusE	1	Loose
antibiotic resistant fusA	1	Loose

Table S4. Genes associated with sporulation in the AL01A genome assembly

Query	KO	Definition
KOJLGGGJ_00003 (416)	K06415	spoVR; stage V sporulation protein R
KOJLGGGJ_00094 (157)	K06405	spoVAC; stage V sporulation protein AC
KOJLGGGJ_00095 (348)	K06406	spoVAD; stage V sporulation protein AD
KOJLGGGJ_00096 (116)	K06407	spoVAE; stage V sporulation protein AE
KOJLGGGJ_00672 (223)	K03091	sigH; RNA polymerase sporulation-specific sigma factor
KOJLGGGJ_00697 (106)	K06283	spoIIID; putative DeoR family transcriptional regulator, stage III sporulation protein D
KOJLGGGJ_00698 (225)	K06386	spoIIQ; stage II sporulation protein Q
KOJLGGGJ_00699 (338)	K06381	spoIID; stage II sporulation protein D
KOJLGGGJ_00724 (231)	K06387	spoIIR; stage II sporulation protein R
KOJLGGGJ_01270 (125)	K02490	spo0F; two-component system, response regulator, stage 0 sporulation protein F
KOJLGGGJ_01280 (499)	K06408	spoVAF; stage V sporulation protein AF
KOJLGGGJ_01282 (526)	K06409	spoVB; stage V sporulation protein B
KOJLGGGJ_01284 (329)	K06413	spoVK; stage V sporulation protein K
KOJLGGGJ_01414 (492)	K06398	spoIVA; stage IV sporulation protein A
KOJLGGGJ_01505 (529)	K06409	spoVB; stage V sporulation protein B
KOJLGGGJ_01604 (645)	K08384	spoVD; stage V sporulation protein D (sporulation-specific penicillin-binding protein)
KOJLGGGJ_01622 (339)	K06383	spoIIGA; stage II sporulation protein GA (sporulation sigma-E factor processing peptidase) [EC:3.4.23.-]
KOJLGGGJ_01623 (211)	K03091	sigH; RNA polymerase sporulation-specific sigma factor
KOJLGGGJ_01624 (259)	K03091	sigH; RNA polymerase sporulation-specific sigma factor
KOJLGGGJ_01795 (158)	K03091	sigH; RNA polymerase sporulation-specific sigma factor
KOJLGGGJ_02045 (178)	K03091	sigH; RNA polymerase sporulation-specific sigma factor
KOJLGGGJ_02069 (67)	K06415	spoVR; stage V sporulation protein R
KOJLGGGJ_02157 (302)	K06402	spoIVFB; stage IV sporulation protein FB [EC:3.4.24.-]
KOJLGGGJ_02181 (334)	K06390	spoIIIAA; stage III sporulation protein AA
KOJLGGGJ_02182 (170)	K06391	spoIIIAB; stage III sporulation protein AB
KOJLGGGJ_02183 (67)	K06392	spoIIIAC; stage III sporulation protein AC
KOJLGGGJ_02184 (128)	K06393	spoIIIAD; stage III sporulation protein AD
KOJLGGGJ_02185 (407)	K06394	spoIIIAE; stage III sporulation protein AE
KOJLGGGJ_02186 (177)	K06395	spoIIIAF; stage III sporulation protein AF
KOJLGGGJ_02187 (154)	K06396	spoIIIAG; stage III sporulation protein AG
KOJLGGGJ_02188 (186)	K06397	spoIIIAH; stage III sporulation protein AH
KOJLGGGJ_02206 (416)	K06399	spoIVB; stage IV sporulation protein B [EC:3.4.21.116]
KOJLGGGJ_02207 (264)	K07699	spo0A; two-component system, response regulator, stage 0 sporulation protein A

KOJLGGGJ_02240 (565)	K13533	kinE; two-component system, sporulation sensor kinase E [EC:2.7.13.3]
KOJLGGGJ_02608 (86)	K06416	spoVS; stage V sporulation protein S
KOJLGGGJ_02639 (404)	K06438	yqfD; similar to stage IV sporulation protein
KOJLGGGJ_02673 (235)	K03091	sigH; RNA polymerase sporulation-specific sigma factor
KOJLGGGJ_02686 (99)	K06412	spoVG; stage V sporulation protein G
KOJLGGGJ_02692 (180)	K04769	spoVT; AbrB family transcriptional regulator, stage V sporulation protein T
KOJLGGGJ_02704 (821)	K06382	spoIIE; stage II sporulation protein E [EC:3.1.3.16]
KOJLGGGJ_02802 (492)	K02491	kinA; two-component system, sporulation sensor kinase A [EC:2.7.13.3]
KOJLGGGJ_02831 (225)	K06384	spoIIM; stage II sporulation protein M
KOJLGGGJ_02837 (114)	K06378	spoIIAA; stage II sporulation protein AA (anti-sigma F factor antagonist)
KOJLGGGJ_02838 (153)	K06379	spoIIAB; stage II sporulation protein AB (anti-sigma F factor) [EC:2.7.11.1]
KOJLGGGJ_02839 (256)	K03091	sigH; RNA polymerase sporulation-specific sigma factor
KOJLGGGJ_02840 (152)	K06405	spoVAC; stage V sporulation protein AC
KOJLGGGJ_02841 (341)	K06406	spoVAD; stage V sporulation protein AD
KOJLGGGJ_02842 (119)	K06407	spoVAE; stage V sporulation protein AE
KOJLGGGJ_02843 (223)	K06407	spoVAE; stage V sporulation protein AE
KOJLGGGJ_02909 (209)	K03091	sigH; RNA polymerase sporulation-specific sigma factor

Table S5. Genes associated with sporulation in the AL05G genome assembly

Query	KO	Definition
IJBEIMBA_00146 (116)	K06407	spoVAE; stage V sporulation protein AE
IJBEIMBA_00147 (348)	K06406	spoVAD; stage V sporulation protein AD
IJBEIMBA_00148 (157)	K06405	spoVAC; stage V sporulation protein AC
IJBEIMBA_00240 (416)	K06415	spoVR; stage V sporulation protein R
IJBEIMBA_00662 (492)	K06398	spoIVA; stage IV sporulation protein A
IJBEIMBA_00792 (329)	K06413	spoVK; stage V sporulation protein K
IJBEIMBA_00794 (526)	K06409	spoVB; stage V sporulation protein B
IJBEIMBA_00796 (499)	K06408	spoVAF; stage V sporulation protein AF
IJBEIMBA_00998 (529)	K06409	spoVB; stage V sporulation protein B
IJBEIMBA_01097 (645)	K08384	spoVD; stage V sporulation protein D (sporulation-specific penicillin-binding protein)
IJBEIMBA_01115 (339)	K06383	spoIIGA; stage II sporulation protein GA (sporulation sigma-E factor processing peptidase) [EC:3.4.23.-]
IJBEIMBA_01116 (211)	K03091	sigH; RNA polymerase sporulation-specific sigma factor
IJBEIMBA_01117 (259)	K03091	sigH; RNA polymerase sporulation-specific sigma factor
IJBEIMBA_01185 (231)	K06387	spoIIR; stage II sporulation protein R
IJBEIMBA_01210 (338)	K06381	spoIID; stage II sporulation protein D
IJBEIMBA_01211 (225)	K06386	spoIIQ; stage II sporulation protein Q
IJBEIMBA_01212 (106)	K06283	spoIIID; putative DeoR family transcriptional regulator, stage III sporulation protein D
IJBEIMBA_01237 (223)	K03091	sigH; RNA polymerase sporulation-specific sigma factor
IJBEIMBA_01424 (158)	K03091	sigH; RNA polymerase sporulation-specific sigma factor
IJBEIMBA_01753 (125)	K02490	spo0F; two-component system, response regulator, stage 0 sporulation protein F
IJBEIMBA_01856 (565)	K13533	kinE; two-component system, sporulation sensor kinase E [EC:2.7.13.3]
IJBEIMBA_01921 (492)	K02491	kinA; two-component system, sporulation sensor kinase A [EC:2.7.13.3]
IJBEIMBA_02063 (67)	K06415	spoVR; stage V sporulation protein R
IJBEIMBA_02246 (302)	K06402	spoIVFB; stage IV sporulation protein FB [EC:3.4.24.-]
IJBEIMBA_02271 (334)	K06390	spoIIIAA; stage III sporulation protein AA
IJBEIMBA_02272 (170)	K06391	spoIIIAB; stage III sporulation protein AB
IJBEIMBA_02273 (67)	K06392	spoIIIAC; stage III sporulation protein AC
IJBEIMBA_02274 (128)	K06393	spoIIIAD; stage III sporulation protein AD
IJBEIMBA_02275 (407)	K06394	spoIIIAE; stage III sporulation protein AE
IJBEIMBA_02276 (177)	K06395	spoIIIAF; stage III sporulation protein AF
IJBEIMBA_02277 (154)	K06396	spoIIIAG; stage III sporulation protein AG

IJBEIMBA_02278 (186)	K06397	spoIIIAH; stage III sporulation protein AH
IJBEIMBA_02296 (416)	K06399	spoIVB; stage IV sporulation protein B [EC:3.4.21.116]
IJBEIMBA_02297 (264)	K07699	spo0A; two-component system, response regulator, stage 0 sporulation protein A
IJBEIMBA_02384 (404)	K06438	yqfD; similar to stage IV sporulation protein
IJBEIMBA_02418 (235)	K03091	sigH; RNA polymerase sporulation-specific sigma factor
IJBEIMBA_02528 (83)	K06416	spoVS; stage V sporulation protein S
IJBEIMBA_02603 (86)	K06416	spoVS; stage V sporulation protein S
IJBEIMBA_02675 (821)	K06382	spoIIE; stage II sporulation protein E [EC:3.1.3.16]
IJBEIMBA_02687 (180)	K04769	spoVT; AbrB family transcriptional regulator, stage V sporulation protein T
IJBEIMBA_02693 (99)	K06412	spoVG; stage V sporulation protein G
IJBEIMBA_02763 (225)	K06384	spoIIM; stage II sporulation protein M
IJBEIMBA_02769 (114)	K06378	spoIIAA; stage II sporulation protein AA (anti-sigma F factor antagonist)
IJBEIMBA_02770 (153)	K06379	spoIIAB; stage II sporulation protein AB (anti-sigma F factor) [EC:2.7.11.1]
IJBEIMBA_02771 (256)	K03091	sigH; RNA polymerase sporulation-specific sigma factor
IJBEIMBA_02772 (152)	K06405	spoVAC; stage V sporulation protein AC
IJBEIMBA_02773 (341)	K06406	spoVAD; stage V sporulation protein AD
IJBEIMBA_02774 (119)	K06407	spoVAE; stage V sporulation protein AE
IJBEIMBA_02775 (223)	K06407	spoVAE; stage V sporulation protein AE
IJBEIMBA_03322 (178)	K03091	sigH; RNA polymerase sporulation-specific sigma factor
IJBEIMBA_03355 (176)	K03091	sigH; RNA polymerase sporulation-specific sigma factor
IJBEIMBA_03378 (78)	K03091	sigH; RNA polymerase sporulation-specific sigma factor
IJBEIMBA_03379 (76)	K03091	sigH; RNA polymerase sporulation-specific sigma factor
IJBEIMBA_03565 (321)	K06398	spoIVA; stage IV sporulation protein A
IJBEIMBA_03631 (134)	K06394	spoIIIAE; stage III sporulation protein AE
IJBEIMBA_03632 (178)	K06395	spoIIIAF; stage III sporulation protein AF
IJBEIMBA_03658 (227)	K06415	spoVR; stage V sporulation protein R
IJBEIMBA_03672 (209)	K03091	sigH; RNA polymerase sporulation-specific sigma factor
